# Transient Neuronal Populations Are Required to Guide Callosal Axons: A Role for Semaphorin 3C

**DOI:** 10.1371/journal.pbio.1000230

**Published:** 2009-10-27

**Authors:** Mathieu Niquille, Sonia Garel, Fanny Mann, Jean-Pierre Hornung, Belkacem Otsmane, Sébastien Chevalley, Carlos Parras, Francois Guillemot, Patricia Gaspar, Yuchio Yanagawa, Cécile Lebrand

**Affiliations:** 1 Department of Cellular Biology and Morphology, University of Lausanne, Switzerland; 2 Inserm, U784, Ecole Normale Supérieure, Paris, France; 3 CNRS, UMR 6216, Developmental Biology Institute of Marseille Luminy, Université de la Méditerranée, Marseille, France; 4 Division of Molecular Neurobiology, National Institute for Medical Research, Mill Hill, London, United Kingdom; 5 Inserm, U839, Institut du Fer à Moulin, Paris, France; 6 Department of Genetic and Behavioral Neuroscience, Gunma University Graduate School of Medicine, Maebashi City, Gunma, Japan; 7 Solution Oriented Research for Science and Technology (SORST), Japan Science and Technology Agency(JST), Saitama, Japan; Cambridge University, United Kingdom

## Abstract

The corpus callosum (CC) is the main pathway responsible for interhemispheric communication. CC agenesis is associated with numerous human pathologies, suggesting that a range of developmental defects can result in abnormalities in this structure. Midline glial cells are known to play a role in CC development, but we here show that two transient populations of midline neurons also make major contributions to the formation of this commissure. We report that these two neuronal populations enter the CC midline prior to the arrival of callosal pioneer axons. Using a combination of mutant analysis and in vitro assays, we demonstrate that CC neurons are necessary for normal callosal axon navigation. They exert an attractive influence on callosal axons, in part via Semaphorin 3C and its receptor Neuropilin-1. By revealing a novel and essential role for these neuronal populations in the pathfinding of a major cerebral commissure, our study brings new perspectives to pathophysiological mechanisms altering CC formation.

## Abstract 2

Neurons, glia, and callosal axons operate as a “ménage à trois” in the development of the corpus callosum.

## Author Summary

The largest commissural tract in the human brain is the corpus callosum, with over 200 million callosal axons that channel information between the two cerebral hemispheres. Failure of the corpus callosum to form appropriately is observed in several human pathologies and can result from defects during different steps of development, including cell proliferation, cell migration, or axonal guidance. Studies to date suggest that glial cells are critical for the formation of the corpus callosum. In this study, we show that during embryonic development, the corpus callosum, which was considered a neuron-poor structure, is in fact transiently populated by numerous glutamatergic and GABAergic neurons. With the use of in vitro graft experiments and of various transgenic mice, we demonstrate that neurons of the corpus callosum are essential for the accurate navigation of callosal axons. Moreover, we discovered that the guidance factor Semaphorin 3C, which is expressed by corpus callosum neurons, acts through the neuropilin 1 receptor to orient axons crossing through the corpus callosum. The present work therefore gives new insights into the mechanisms involved in axon guidance and implies that transient neurons work together with their glial partners in guiding callosal axons.

## Introduction

The largest commissural tract in the human brain is the corpus callosum (CC), with over 200 million axons that act as a conduit for information between the two cerebral hemispheres. Callosally projecting neurons are Satb2-positive pyramidal projection neurons positioned, in rodents, in upper and lower cortical layers and that extend their axons through the CC [1]–[4]. More than 50 human syndromes result in agenesis of the CC (AgCC) and have an associated genetic etiology [5],[6]. AgCC can result from defects during different steps of callosal development, including cell proliferation, migration, or a failure in axonal guidance within the CC [6].

Studies to date suggest that a specialized population of glia adjacent to the midline are central for the formation of the CC [7]–[13]. The primitive astroglial cells of the “glial” sling form a bridge-like structure at the midline between the two lateral ventricles and are required for the development of the CC [12],[14],[15]. Additional glial structures in the CC were described: radial glial cells in the glial wedge (GW) and astrocytes in the indusium griseum (IG) [6],[7],[10]. Recent observations in mice and humans showed that many neurons are also present within the “glial” sling [16],[17]. Similarly, scattered neurons were observed within the cat CC during early postnatal life [18],[19]. However, whether these populations have a specific function during development has not been investigated.

In this paper, we characterize the embryonic midline cellular organization at times prior to and during the formation of the CC. Through this effort, we discovered that in mouse embryos, uncharacterized GABAergic neurons intermix with glutamatergic neurons within the entire CC white matter. Here, we explore the possibility that these populations act in conjunction with midline glial cells to mediate the formation of the CC. We first investigated the identity of these populations and their spatial organization relative to ingrowing callosal axons. To determine whether these populations are functionally important, we examined the consequences of genetic ablation of a subpopulation of neurons as well as testing whether neuronal cells of the CC contribute to axonal guidance there. We show that the two neuronal populations that transiently populate the CC form a complex cellular network and that CC GABAergic interneurons are required for the proper organization of this network. Furthermore, ex vivo and in vitro experiments indicate that GABAergic and glutamatergic neurons of the CC are able to attract callosal axons.

With regards to the signaling pathways that contribute to the formation of the CC, a number of studies have demonstrated that midline glial cells are the principal CC guidepost cells and secrete guidance factors that channel the callosal axons into the correct path [7]–[13]. These guidance signaling factors include Netrin1/DCC, Slit2/Robo1, ephrins/Eph, Semaphorin/Neuropilin-1 (Npn-1), and Wnt [5],[6],[20]–[28]. Mutant mice for these guidance cues and their receptors exhibit callosal defects that range from minor, with few axons leaving the callosal track, to severe, with complete AgCC.

Although the Semaphorin/Npn-1 signaling had been shown to be essential for CC development, the specific semaphorin ligand involved in this process, its source within the midline, as well as its precise function, remained to be determined [26],[29]. In this study, we show that the transient population of CR-positive glutamatergic neurons expresses Sema3C and that either the ectopic transplantation of glutamatergic neurons or the ectopic expression of this ligand is sufficient to attract callosal axons. The use of *Sema3C* knockout (KO) mice confirms a novel and essential role of this factor in the pathfinding of callosal axons. Taken together, these results reveal that transient GABAergic and glutamatergic neurons are required for the formation of the CC. The present work, therefore, gives new insights into the mechanisms involved in axon guidance and implicates that transient neuronal populations work in conjunction with their glial partners in the guidance of callosal axons.

## Results

### Glutamatergic and GABAergic Neurons Populate the Neonatal CC

**Figure 1:**
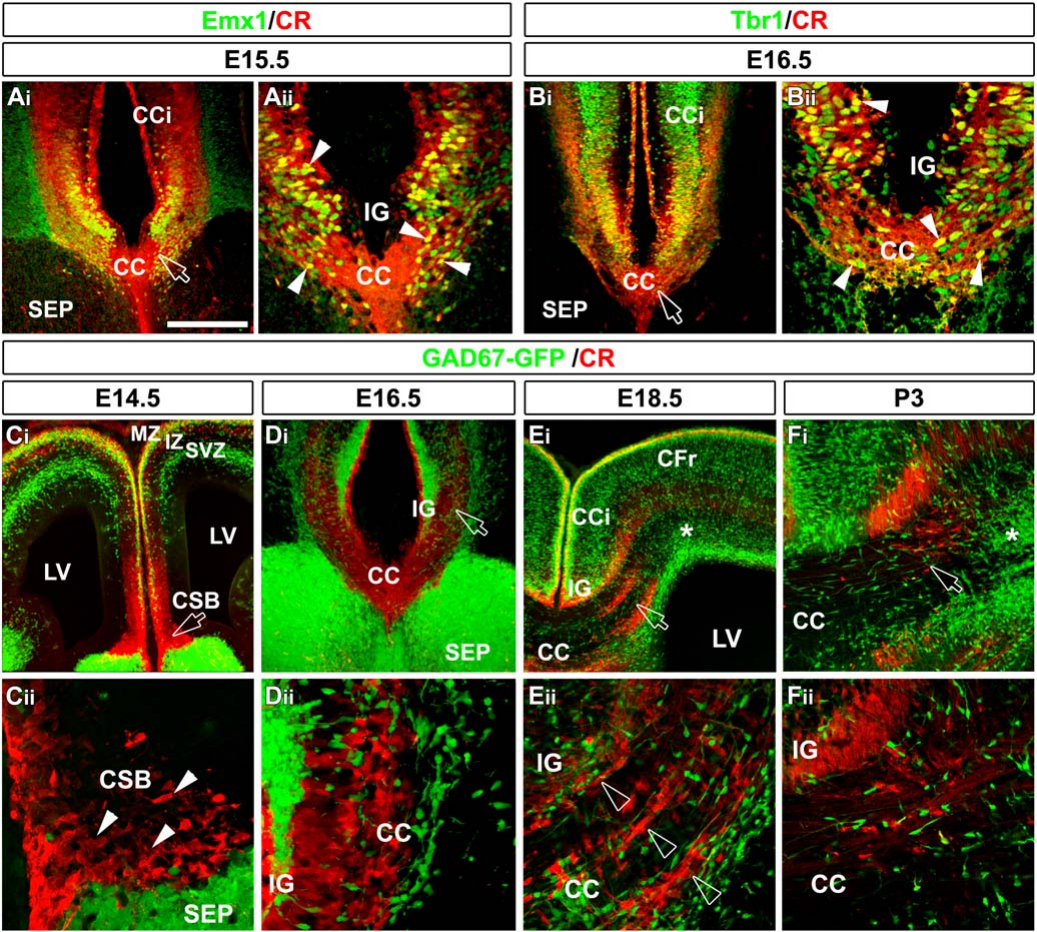
Nature and organization of CR and of GAD67/Mash1-GFP–expressing neurons in the CC during development. (A and B) Double immunohistochemistry for CR and Emx1 (A_i_–A_ii_) and for CR and Tbr1 (B_i_–B_ii_) in coronal sections from mice at E15.5 (A_i_–A_ii_) and E16.5 (B_i_–B_ii_). (A_ii_ and B_ii_) are higher power views of a single confocal plan of the CC *Z*-stack seen in (A_i_ and B_i_), respectively (open arrows). The CR^+^ neurons of the CC are glutamatergic in nature since they express Emx1 and Tbr1. A yellow channel highlights the colocalization of markers. (C–F) Immunohistochemical staining for CR (in red) in coronal sections from GAD67-GFP transgenic mice at E14.5 (C_i_–C_ii_), E16.5 (D_i_–D_ii_), E18.5 (E_i_–E_ii_), and P3 (F_i_–F_ii_). (C_ii_, D_ii_, E_ii_, and F_ii_) are higher power views of a single confocal plane of the CC *Z*-stack seen in (C_i_, D_i_, E_i_, and F_i_), respectively (open arrows). (C_i_–C_ii_) At E14.5, CR^+^ neurons are located at the corticoseptal boundary (CSB) (arrowheads in [C_ii_]), whereas GAD67-GFP^+^ neurons are present in the subventricular zone (SVZ), IZ, and subplate of the frontal cortex. (D_i_–D_ii_) At E16.5, GAD67-GFP^+^ neurons start to intermix with CR^+^ neurons in one compact strip in the midline and lateral CC. (E_i_–E_ii_) At E18.5, although the CC has undergone its dorsoventral compartmentalization, the CR^+^ neurons segregate into three distinct compact strips (open arrowheads) in the lateral extension of the CC, whereas GAD67-GFP^+^ neurons are dispersed in the entire CC white mater. In addition, GAD67-GFP^+^ neurons form a cluster of cells in the extreme lateral part of the CC (*). (F_i_–F_ii_) At P3, CR^+^ neurons have nearly disappeared from the CC, while GFP^+^ neurons are still present. Bar indicates 435 µm in (C_i_ and E_i_), 220 µm in (A_i_, B_i_, D_i_, and F_i_), 110 µm in (A_ii_, B_ii_, E_ii_, and F_ii_), and 70 µm in (C_ii_ and D_ii_). CFr, frontal cortex; LV, lateral ventricle; MZ, marginal zone.

Previous work has implicated the “glial” sling as central for the establishment of the CC [11],[12]. Despite its name, the sling has been shown to contain at least one neuronal population whose function to date is undetermined [17]. As a starting point for investigating whether this neuronal population, and perhaps others, contribute to the formation of the CC, we undertook an immunological analysis of this region during embryonic and postnatal development. In addition to glial cells, upon immunostaining with antibodies against βIII-tubulin, MAP2A, and NeuN, we detected a large number of neurons, not only within the “glial” sling, but also within the entire white matter of the developing CC from embryonic day 12.5 (E12.5) until postnatal day 14 (P14). In particular, our molecular analysis revealed two distinct neuronal subpopulations. One that includes the population previously described as “sling neurons” was comprised of a population of differentiated glutamatergic neurons (Figure 1). We found that this population expressed the homeobox transcription factor Emx1 and T-box transcription factor Tbr1, which are known to promote glutamatergic fate [30]–[32], the type 1 vesicular glutamate transporter (VGLUT1), and the calcium binding protein calretinin (CR) (Figure 1A_i_–1A_ii_ to 1B_i_–1B_ii_ and unpublished data). Nearly all the CR-positive embryonic neurons of the CC intermediate zone (IZ) coexpressed the glutamatergic marker Tbr1 (91.7±1.3% at E16.5, *n* = 1,415) (Figure S1A_i_). The other population was composed of GABAergic interneurons and was identified using either: i) a *GAD67-GFP* mouse line in which the green fluorescent protein (GFP) is reliably expressed within GABAergic neurons [33] (Figure 1C_i_–1C_ii_ to 1F_i_–1F_ii_) or ii) a *Mash1-GFP* transgenic mouse line (*Ascl1*, Mammalian achaete-scute homolog) (GENSAT) that labels telencephalic GABAergic interneurons derived from *Mash1*-expressing progenitors of the ventral telencephalon [34],[35] (Figure S1C_i_–S1C_ii_, S1D_i_–S1D_ii_ and Figure S4E_i_). A careful analysis of the colocalization between CR and GAD67-GFP in the neurons of the CC IZ indicates that these two neuronal population are exclusive at embryonic ages (0.7±0.2% at E16.5, *n* = 1,907; and 1.4±0.4%, *n* = 1,898 at E18.5) (Figure 1C_i_–1C_ii_ to 1E_i_–1E_ii_ and Figure S1B_i_). The two neuronal population identified by the expression of CR or GAD67-GFP included half of the CC IZ cells at embryonic ages (46% at E16.5, *n* = 5,561; 53% at E18.5, *n* = 6,495). At E16.5, the CR-positive glutamatergic neurons constitute 73.6±0.012% of these neurons, whereas the GAD67-GFP–positive GABAergic interneurons constitute 25.9±0.012% (*n* = 2,580; Figure S1B_ii_). At E18.5, the proportion of both neuronal cell types is even (51.0±0.026% for CR^+^ neurons, 48.3±0.025% for GAD67-GFP^+^ neurons, *n* = 3,442; Figure S1B_iii_). As expected, the CR-positive glutamatergic neurons and GABAergic interneurons did not express any of the glial markers nestin, GLAST, and GFAP (Figure S1C_i_–S1C_ii_ to S1E_i_–S1E_vi_ and unpublished data) previously found on early astroglial cells of the CC, IG, and GW [10].

### Transient CC Neuronal Populations Are Located along the Path of Callosal Axons

We wished to determine whether CR-positive glutamatergic and GAD67/Mash1-GFP–positive GABAergic neurons are present at times and in a spatial distribution consistent with their contribution to the formation of CC axonal paths. As such, we undertook a longitudinal analysis of these populations during development to establish their relationship to callosal axons.

Between E12.5 and E15.5, the glutamatergic CR-positive neurons occupy a strategic midline position at the corticoseptal boundary (CSB) (Figure 1C_i_–1C_ii_, arrowheads). As such, they occupy this area prior to any callosal axons entering this region (Figure S2A_i_–S2A_iv_). At this stage, the GAD67/Mash1-GFP–positive interneurons are still migrating within the marginal zone, subplate, IZ, and subventricular zone of the frontal cortical area (Figure 1C_i_).

**Figure 2:**
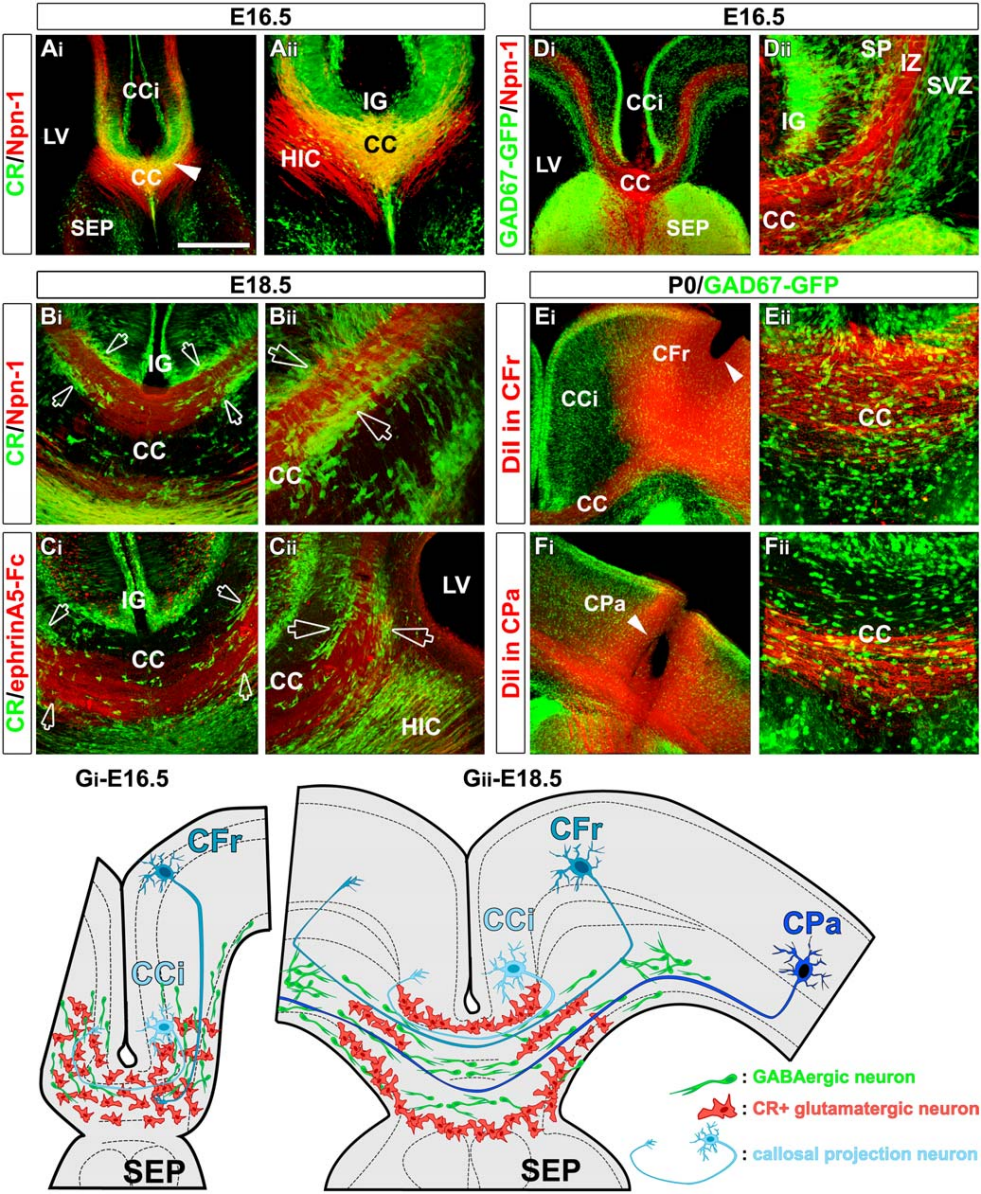
CR-positive glutamatergic neurons and GAD67/Mash1-GFP-positive interneurons segregate callosal projections. (A–C) Double immunohistochemical staining for CR and Npn-1 (A_i_–A_ii_ and B_i_–B_ii_) and for CR and ephrinA5 binding sites (C_i_–C_ii_) in coronal CC sections from E16.5 (A_i_–A_ii_) and E18.5 (B_i_–B_ii_ and C_i_–C_ii_) mice. (A_ii_) is a high-power view of the CC seen in (A_i_). (B_ii_ and C_ii_) illustrate the lateral extensions of the CC seen respectively in (B_i_ and C_i_). (A_i_–A_ii_) At E16.5, the CR^+^ glutamatergic neurons extend throughout the entire CC and surround the pioneer axons that start to cross the midline (arrowhead in [A_i_]). (B_i_–B_ii_ and C_i_–C_ii_) At E18.5, the CR^+^ glutamatergic neurons segregate in three strips (open arrows) that delineate the dorsoventral pathways labeled for Npn-1 or ephrin-A5 binding sites. (D_i_–D_ii_) Immunohistochemistry for Npn-1 in coronal telencephalon slices from E16.5 GAD67-GFP transgenic mice. (D_ii_) is a high-power view of the CC seen in (D_i_). At E16.5, callosal axons encounter GAD67-GFP^+^ GABAergic interneurons while they are migrating tangentially through the IZ. (E_i_–E_ii_ and F_i_–F_ii_) P0 CC coronal section of GAD67-GFP transgenic mice showing axonal tracing of callosal axons by insertion of DiI crystals, respectively, in the frontal (CFr, arrowhead in [E_i_]) and parietal (CPa, arrowhead in [F_i_]) cortices. (E_ii_ and F_ii_) are high-power views of the corresponding sections in (E_i_ and F_i_), respectively. Callosal axons from the medial cortical area grow in the dorsal path of the CC, whereas axons from the lateral cortex grow in the ventral path. GAD67-GFP^+^ GABAergic interneurons are dispersed in the entire CC and surround callosal axon bundles without delineating the dorsoventral paths. (G_i_–G_ii_) Schematic drawing representing the complementary organization of CR^+^ glutamatergic neurons (in red) and of GAD67/Mash1-GFP GABAergic interneurons (in green) in the CC at E16.5 (G_i_) and E18.5 (G_ii_). The pioneer callosal axons originating from the cingulate (CCi) cortex, as well as the later-growing callosal axons from the frontal and the parietal cortices are represented in blue and are seen to navigate through the CC neurons. Bar indicates 435 µm in (A_i_, D_i_, E_i_, and F_i_), 220 µm in (A_ii_, B_i_, C_i_, and C_ii_), 110 µm in (D_ii_, E_ii_, and F_ii_), and 70 µm in (B_ii_). HIC, hippocampal commissure; LV, lateral ventricle; SP, subplate.

At E16.5, GAD67/Mash1-GFP neurons intermix with the CR neurons at the midline and in the lateral part of the CC (Figure 1D_i_–1D_ii_). At this age, CC pioneer callosal axons start to cross the midline [36],[37], whereas later-growing axons originating from the frontal cortex just approach the lateral border of the CC (Figure S2B_i_–S2B_ii_ and S2C_i_–S2C_ii_). All these axons expressing the transmembrane receptor Npn-1 come into contact with both the CR-positive glutamatergic neurons (Figure 2A_i_–2A_ii_ and Figure 2G_i_) and GAD67/Mash1-GFP–positive interneurons (Figure 2D_i_–2D_ii_ and Figure 2G_i_) while growing through the CC.

From E18.5 to P3, CR-positive neurons are positioned topographically in three stripes within anatomically distinct regions of the main body of the CC (Figure 1E_i_–1E_ii_, open arrowheads). They were located: i) at the border of the IG and of the cingulate cortex (CCi), ii) in the middle of the white matter of the CC, and iii) in the “glial” sling at the border of the septum (SEP) and in the region of the GW. The three stripes of glutamatergic CR-positive neurons delineate both a ventral and a dorsal CC axonal path (Figure 2B_i_–2B_ii_, Figure 2C_i_–2C_ii_, open arrows, and Figure 2G_ii_). Labeling using carbocyanine dyes showed that these dorsal and ventral axonal paths originate from distinct mediolateral cortical areas (Figure 2E_i_–2E_ii_, Figure 2F_i_–2F_ii_, and Figure S2D_i_–S2D_iv_). This dorsoventral organization was further delineated by the restricted expression pattern of receptors for axon guidance molecules such as Npn-1 (Figure 2B_i_–2B_ii_ and Figure S2F) and Deleted in Colorectal Cancer (DCC) (Figure S2H) dorsally, and ephrinA5 binding sites ventrally (Figure 2C_i_–2C_ii_ and Figure S2G). By contrast, at this age, GAD67/Mash1-GFP–positive neurons are more diffusely distributed within the entire white matter of the CC and are seen surrounding the growing commissural axons (Figure 1E_i_–1E_ii_, Figure 2E_i_–2E_ii_, and 2F_i_–2F_ii_).

Glutamatergic neurons of the CC expressing CR disappeared abruptly between P1 and P3, whereas GAD67/Mash1-GFP GABAergic neurons disappeared progressively in a spatiotemporal gradient, from P7 at the midline, until P21 in the extreme lateral part of the CC (Figure 1F_i_–1F_ii_ and Figure S3A_i_–S3A_ii_ to S3D_i_–S3D_ii_). Cleaved caspase 3 staining and ultrastructural changes showed that both neuronal populations of the CC died at early postnatal ages (Figure S3). Our ultrastructural study revealed that dying neurons adopt different morphological types: a non-lysosomal vesiculate type (type IIIB) for GAD67-GFP–positive GABAergic interneurons or an autophagic type (type II) for glutamatergic neurons [38].

Our results, therefore, demonstrate that the CC is more heterogeneous than previously thought. Specifically, the entire CC white matter contains transient CR-positive glutamatergic neurons and GABAergic interneurons that correspond to the organization of the callosal projections within this region. Furthermore, their location and the timing of their appearance raise the possibility that these neurons actively participate in the guidance of callosal axons.

### Topographic Positioning between the Glutamatergic and GABAergic Neurons, and the Developing Callosal Axons

To study the spatial relationships between CC neurons and callosal axons, we used electron microscopy and 3-D analysis of high-resolution confocal image stacks.

Electron microscopy and pre-embedding immunocytochemistry showed CC neurons apposed to one another, forming a complex cellular network (stars) around callosal axons (arrowheads) (Figure S4A to S4D_i_–S4D_ii_ and unpublished data). To determine how glutamatergic CR-positive and GABAergic GAD67/Mash1-GFP–positive neurons participate in this cellular network, we generated isosurface maps (Figure S4E_ii_) using immunostaining to label both neuronal populations and cell nuclei (CR, GFP, Hoechst) (Figure S4E_i_). Isosurface reconstructions allowed us to explore the geometry of this cellular organization using the navigator function of IMARIS 4.3 software (Figure S4E_iii_–S4E_vii_ and Video S1). The 3-D visualization showed that both CR-positive glutamatergic neurons and GAD67/Mash1-GFP interneurons contributed in forming the “walls” of a complex cellular network surrounding callosal axons inside the CC (Figure S4E_iii_–S4E_vii_ and Video S1). Our observations thus indicate that the two neuronal populations that transiently populate the CC form a dense cellular network that interacts intimately with the growing commissural axons.

### CC Formation Is Impaired in *Mash1* Mutant Mice Lacking GABAergic Interneurons

**Figure 3:**
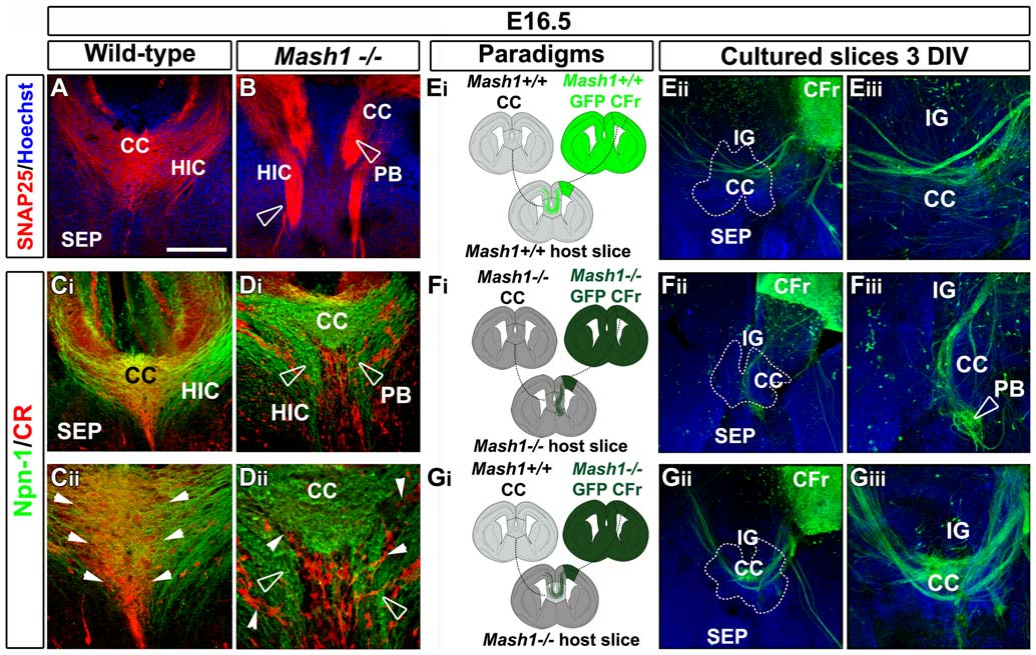
CC neuronal network integrity is important for pathfinding by callosal axons. (A–D) Single immunohistochemistry for SNAP25 (A and B) and double immunohistochemistry for Npn-1 and CR (C_i_–C_ii_ and D_i_–D_ii_) in coronal CC sections from E16.5 WT (A, C_i_, and C_ii_) and *Mash1^−/−^* (B, D_i_, and D_ii_) mice. (C_ii_ and D_ii_) are higher power views of the medial CC seen in (C_i_ and D_i_). (A, C_i_, and C_ii_) At E16.5, both callosal and hippocampal commissure (HIC) fibers labeled with SNAP25 or Npn-1 begin to cross the midline and grow towards the contralateral cortex. (B, D_i_, and D_ii_) By contrast, in *Mash1^−/−^* brains, the majority of callosal and hippocampal fibers do not cross the midline and form large ectopic bundles of axons on either side of it, reminiscent of Probst bundles (PB; open arrowheads). (D_i_ and D_ii_) The loss of Mash1-positive GABAergic interneurons of the CC causes the disorganization of glutamatergic CR^+^ neurons at the midline (compare [D_ii_] with [C_ii_], arrowheads). (E_i_) Experimental paradigm used to confirm the growth of E16.5 GFP^+^ WT callosal axons in CC transplants and slices from WT mice. (E_ii_–E_iii_) GFP immunocytochemistry showing that WT GFP^+^ callosal axons grow normally and cross the midline when they are confronted with a WT environment. (F_i_) Experimental paradigm used to confirm the growth defects of E16.5 GFP^+^ *Mash1* mutant callosal axons in CC transplants and slices from *Mash1^−/−^* mice. (F_ii_–F_iii_) GFP immunocytochemistry showing that GFP^+^ callosal axons of *Mash1^−/−^* cortical explants do not cross the midline, but rather form Probst bundles (PB, open arrowhead). (G_i_) Experimental paradigm used to test whether the CC neuronal network integrity is necessary and sufficient to direct the growth of callosal axons. To this end, WT CC is transplanted in a Mash1^−/−^ slice. (G_ii_–G_iii_) GFP immunocytochemistry showing the complete restoral of Mash1^−/−^ callosal axons pathfinding. Dashed lines outline the CC transplant localizations. Brain slices in (E_ii_–E_iii_, F_ii_–F_iii_, and G_ii_–G_iii_) were counterstained with Hoechst. Bar indicates 435 µm in (E_ii_, F_ii_, and G_ii_), 220 µm in (A, B, C_i_, D_i_, E_iii_, F_iii_, and G_iii_), and 110 µm in (C_ii_ and D_ii_). CFr, frontal cortex.

Given the density and complexity of the neuronal network that we identified, it is relatively difficult to unravel its function in CC formation. As a first step, we analyzed the brains of mutant mice defective for the production of GABAergic interneurons. *Mash1* is a transcription factor expressed in GABAergic progenitors of the ventral telencephalon and its inactivation severely impairs the production of cortical interneurons [34],[35]. Consistently, we found that the CC of *Mash1* mutant embryos was nearly devoid of GABAergic interneurons (GABA-positive neurons: 1.390±0.146 neurons/mm^2^ in CC of wild-type (WT) mice versus 0.167±0.055 neurons/mm^2^ in CC of *Mash1^−/−^* mice, *p*<0.001) (compare Figure S5C_i_–S5C_ii_ with S5D_i_–S5D_ii_). To investigate how the lack of GABAergic interneurons affects CC formation, we also examined whether the other CC cell types are impaired by *Mash1* inactivation. At E16.5, the CC glial cells' localization, morphology, and expression of guidance factors (ephrins, semaphorins, and Slit2) were not affected in *Mash1* mutant embryos (Figure S5E_i_–S5E_ii_ and S5F_i_–S5F_ii_, and Figure S6). By contrast, some glutamatergic CR-positive neurons were displaced ventrally at the midline (compare Figure 3C_i_–3C_ii_ and 3D_i_–3D_ii_, arrowheads). Thus, in *Mash1* mutants, although the glial scaffold appears normal, the CC neuronal network is severely affected, with a lack of GABAergic interneurons and a displacement of CR-positive glutamatergic neurons at the midline.

Our analysis showed that *Mash1* inactivation leads to major alterations of axonal paths in the CC (compare Figure 3A with 3B, compare Figure 3C_i_–3C_ii_ with 3D_i_–3D_ii_, and see Figure S5G_i_–S5G_v_ with S5H_i_–S5H_v_). From E16.5 to E18.5, *Mash1^−/−^* embryos exhibited partial (Figure 3D_i_–3D_ii_ and Figure S6) to complete (Figure 3B and Figure S5H_i_–S5H_iv_) AgCC, with few axons, if any, crossing the midline. Although axons were impaired in midline crossing, they expressed normal levels of L1, Npn-1, and DCC guidance receptors (unpublished data, Figure 3D_i_–3D_ii_, and Figure S6G_i_–S6G_ii_ and S6H_i_–S6H_ii_). Instead, callosal axons entered the IG or the SEP and formed two large ectopic fascicles known as Probst bundles that are characteristic of acallosal mammalian forebrains (Figure 3B and Figure 3D_i_–3D_ii_, open arrowheads). In addition, DiI-labeled axons that are normally located in the dorsal and ventral paths of the CC intermingle in *Mash1^−/−^* embryos before reaching the midline (compare Figure S5G_i_–S5G_v_ and S5H_i_–S5H_v_). On the other hand, in *Mash1^−/−^*, the area specification of the dorsal telencephalon [39], and the laminar distribution of the Tbr1-positive cortical layers V–VI that contain pyramidal callosal neurons and of Sabtb2-positive callosally projecting neurons were normal (unpublished data, and compare Figure S5A_i_–S5A_ii_ and S5B_i_–S5B_ii_).

**Figure 4:**
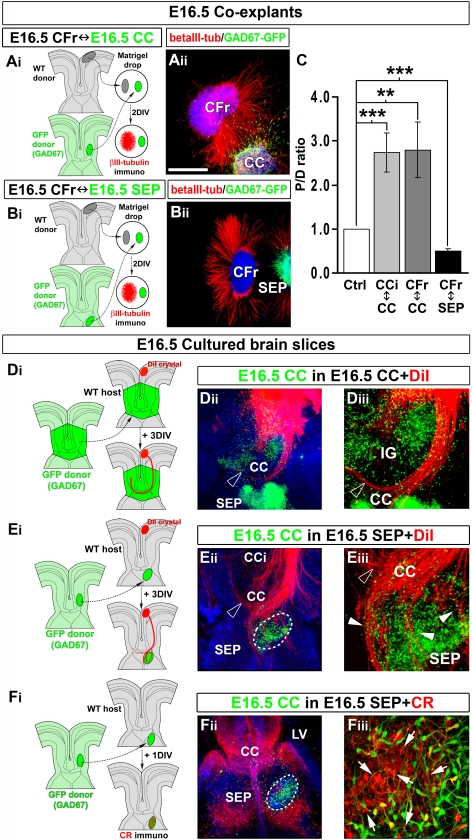
CC region enriched in both types of neurons attracts callosal axons. (A–C) Experimental paradigms used to determine whether the CC (A_i_) and the SEP (B_i_) exert attractive or repulsive effects on cortical axons. (A_ii_ and B_ii_) Immunohistochemical staining for βIII-tubulin on E16.5 frontal cortex (CFr) cocultured with E16.5 GAD67-GFP^+^ CC (A_ii_) or SEP (B_ii_). (C) Quantification of the axonal guidance responses to E16.5 CC and SEP explants. Data are expressed as a *P/D* ratio, where *P* and *D* are the mean fluorescence ±SEM intensity in the quadrants proximal and distal to the CC or SEP explants. Double asterisks (**) indicate significantly different with *p*<0.01; triple asterisks (***) indicate significantly different with *p*<0.001. CC neurons cause strong chemoattraction on cingulate (CCi) and frontal axons, whereas the SEP is repulsive. (D_i_) In vitro model of organotypic slices at E16.5 used to study callosal axon navigation through CC after transplantation of E16.5 CC and surrounding regions. (D_ii_–D_iii_) GAD67-GFP^+^ neurons maintain their initial organization through the explant after 3 d in vitro. DiI-labeled callosal axons grow normally and cross the CC midline within the large GAD67-GFP^+^ CC explant. (E_i_) Experimental paradigm used to determine whether the CC neurons exert attractive effects on callosal axons. To test this hypothesis, a small explant of E16.5 GAD67-GFP^+^ lateral CC containing GABAergic and glutamatergic neurons is transplanted in the nonpermissive septal region. (E_ii_–E_iii_) DiI staining showing that callosal axons penetrate the SEP toward the CC explant enriched in both type of neurons. (F_i_–F_iii_) CR immunostaining showing that E16.5 lateral CC explants obtained from GAD67-GFP transgenic mice contained CR-positive glutamatergic neurons (arrows) in addition to GABAergic interneurons. Brain tissues in (A_ii_, B_ii_, D_ii_, E_ii_, and F_ii_) were counterstained with Hoechst. Bar indicates 485 µm in (B_ii_), 435 µm in (A_ii_, D_ii_, E_ii_, and F_ii_), 220 µm in (D_iii_ and E_iii_), and 70 µm in (F_iii_). LV, lateral ventricle.

To investigate whether this severe axon guidance phenotype was due to defects in the CC region rather than to altered development of other regions in the *Mash1* mutants, we performed transplantations of the CC into E16.5 telencephalic slices, using different combinations of WT and *Mash1^−/−^* embryos (Figure 3 and Figure S7). In our slice assays, as in in vivo [36],[37], the callosal axons from dorsolateral neocortex develop later than pioneer axons, and after E16.5, their growth cones enter the CC region in successive streams over a period of several days (Figure S2B_i_–S2B_ii_ to S2C_i_–S2C_ii_). When dorsal cortical explants from GFP-positive *Mash1^−/−^* mice were transplanted into WT slices (*n* = 7 out of 7), GFP-labeled callosal axons crossed the midline, whereas transplantations of dorsal cortex from GFP-positive WT mice into *Mash1^−/−^* slices (*n* = 6 out of 6) lead to an impairment in axonal midline crossing (Figure S7). These experiments suggested that callosal axons mistargeting in *Mash1* mutant embryos is due to defects in CC midline and surrounding structures. To further investigate this issue, we then performed reversion experiments (Figure 3). When dorsal cortical explants from GFP-positive WT mice and explants of the CC region from WT donors were transplanted into a WT brain slice, a majority of GFP-labeled callosal axons crossed the midline (Figure 3E_i_–3E_iii_; *n* = 5 out of 6), thereby reproducing the in vivo behavior of callosal axons. By contrast, with GFP-positive *Mash1^−/−^* cortical and *Mash1^−/−^* CC explants transplanted into *Mash1^−/−^* slices, GFP-positive callosal axons failed to cross the midline (Figure 3F_i_–3F_iii_; *n* = 3 out of 3). We then tested whether the transplantation of WT CC into *Mash1^−/−^* mutant slices could restore correct pathfinding of GFP-positive *Mash1^−/−^* callosal axons (Figure 3G_i_–3G_iii_). Remarkably, WT CC restored normal axonal guidance of the majority of *Mash1^−/−^* callosal axons, but only when the transplant comprised the medial and lateral parts of the CC that contain the GABAergic interneuron population we have identified (Figure 3G_i_–3G_iii_; *n* = 4 out of 6). Transplantation experiments of GAD67-GFP–positive WT CC into WT slices confirmed that CC GABAergic interneurons remain through the CC transplant and maintained their initial organization after several days in vitro (Figure 4D_i_–4D_iii_; *n* = 4 out of 4). Therefore, callosal axons misrouting observed in *Mash1* mutant embryos is largely due to defects in the CC region.

Altogether, our experiments indicate that *Mash1* inactivation does not impair callosal pyramidal neurons differentiation but leads to a severe modification of the CC neuronal network. These results suggest that CC GABAergic interneurons, which are lacking in *Mash1^−/−^* mice, may participate in callosal axons guidance and support the idea that the integrity of this neuronal CC network is important for normal callosal axons navigation.

### Chemoattractive Activity of the Two CC Neuronal Populations

**Figure 5:**
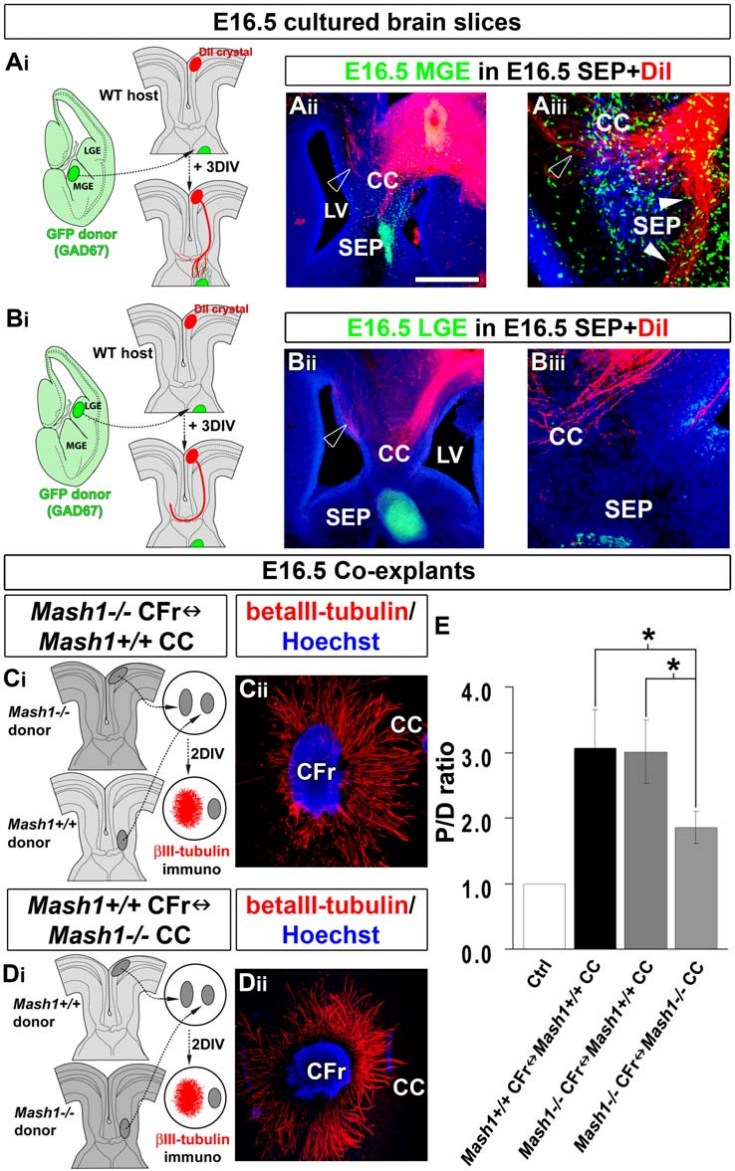
GABAergic interneurons exert part of the attractive influence on callosal axons. (A_i_) Experimental paradigm used to determine whether CC GABAergic interneurons attract callosal axons. To test this hypothesis, we placed a small explant of E16.5 medial ganglionic eminence (MGE) that generates the CC GABAergic interneurons into the SEP of an E16.5 slice. (A_ii_–A_iii_) DiI staining showing that numerous callosal axons penetrate through the SEP and are attracted by GAD67-GFP^+^ migrating GABAergic interneurons derived from the MGE explant. (B_i_) As a control, E16.5 GAD67-GFP^+^ lateral ganglionic eminence (LGE) explant that does not generate CC GABAergic interneurons is transplanted into the SEP of an E16.5 slice. (B_ii_–B_iii_) LGE explant does not attract callosal axons through the nonpermissive septal region. Open arrowhead in (A_ii_ and B_ii_) highlights callosal axons crossing the midline of the CC, whereas arrowheads in (A_iii_) indicate callosal axons that are misrouted within the SEP. Brain slices in (A_ii_, A_iii_, B_ii_, and B_iii_) were counterstained with Hoechst. (C_i_) Experimental paradigm used to analyze whether *Mash1^−/−^* cortical axons respond normally to CC attraction. (C_ii_) Immunohistochemical staining for βIII-tubulin on E16.5 *Mash1^−/−^* frontal cortex (CFr) cocultured with E16.5 *Mash1^+/+^* CC. (D_i_) Experimental paradigm used to analyze whether CC GABAergic interneurons promote cortical axons attraction. For this, WT CFr explants were cocultured with small explants of *Mash1^−/−^* CC missing GABAergic interneurons (D_ii_) Immunohistochemical staining for βIII-tubulin on E16.5 *Mash1^+/+^* frontal cortex cocultured with E16.5 *Mash1^−/−^* CC. (E) Quantification of the axonal guidance responses to CC explants. Data are expressed as a *P/D* ratio ±SEM, where *P* and *D* are the mean fluorescence intensity in the quadrants proximal and distal to *Mash1^+/+^* or *Mash1^−/−^* CC explants. An asterisk (*) indicates significantly different with *p*<0.05. *Mash1^−/−^* cortical axons are normally attracted by WT CC. By contrast, *Mash1* mutant CC is still attractive for cortical axons, but with a reduced efficiency compare to WT CC. Bar indicates 485 µm in (A_ii_, B_ii_, C_ii_, and D_ii_) and 120 µm in (A_iii_ and B_iii_). LV, lateral ventricle.

**Figure 6:**
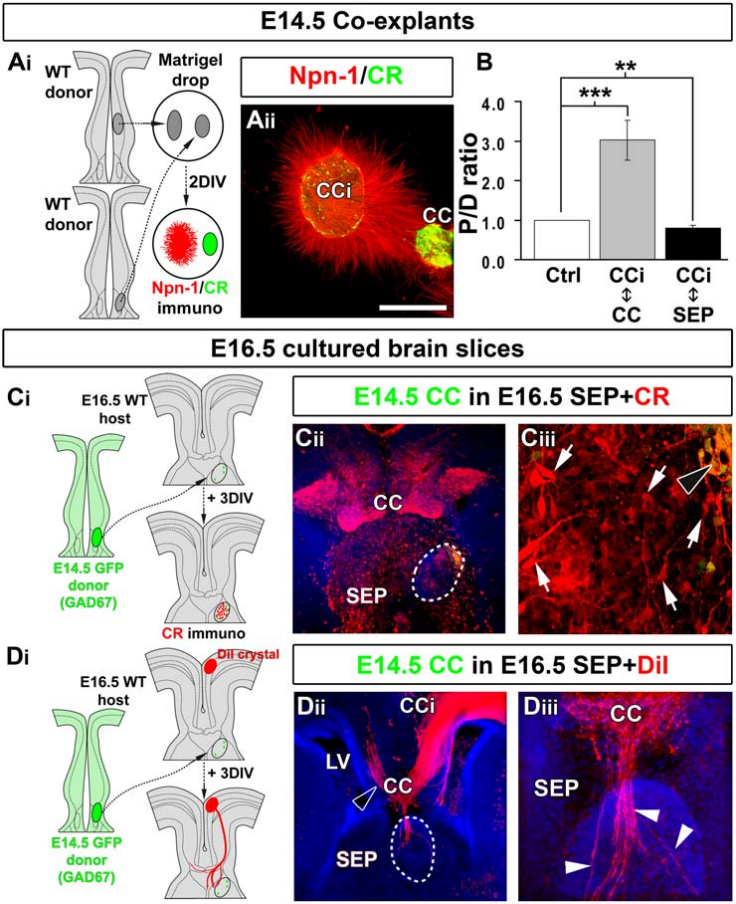
CC region enriched in glutamatergic CR-positive neurons attracts callosal axons. (A_i_) Experimental paradigm used to determine whether the glutamatergic CR-positive neurons of the CC exert attractive effects on cortical axons. (A_ii_) Immunohistochemical staining for CR/Npn-1 on coexplants of E14.5 cingulate cortex (CCi) cocultured with E14.5 CC. (B) Quantification of the axonal guidance responses to E14.5 CC and septum (SEP) explants. Data are expressed as a *P/D* ratio, where *P* and *D* are the mean fluorescence intensity ±SEM in the quadrants proximal and distal to the CC or SEP explants. Double asterisks (**) indicate significantly different with *p*<0.01; triple asterisks (***) significantly different with *p*<0.001. Glutamatergic CR-positive neurons of the CC cause strong chemoattraction on cingulate axons, whereas the SEP is repulsive. (C_i_–C_iii_) CR immunostaining showing that E14.5 CC midline explants obtained from GAD67-GFP transgenic mice are highly enriched in CR-positive glutamatergic neurons (arrows) compared to GAD67-GFP^+^ GABAergic interneurons (black arrowhead). (D_i_) Experimental paradigm used to determine whether the CC glutamatergic neurons exert attractive effects on callosal axons. To test this hypothesis, a small explant of E14.5 CC containing glutamatergic neurons but no GAD67-GFP–positive GABAergic interneurons is transplanted in the nonpermissive septal region. (D_ii_–D_iii_) DiI staining showing that numerous callosal axons penetrate the SEP through the CC explant containing only CR-positive glutamatergic neurons. Open arrowhead in (D_ii_) highlights callosal axons crossing the midline of the CC, whereas arrowheads in (D_iii_) indicate callosal axons that are misrouted within the SEP through the explant. Dashed lines outline the transplant localizations. Brain slices in (C_ii_, D_ii_, and D_iii_) were counterstained with Hoechst. Bar indicate 485 µm in (C_ii_ and D_ii_), 435 µm in (A_ii_), 120 µm in (D_iii_), and 70 µm in (C_iii_). LV, lateral ventricle.

To further understand how CC neurons contribute to callosal axon navigation, we tested whether a CC region enriched in GABAergic and/or CR-positive glutamatergic neurons could promote the growth of callosal axons in coexplant and heterotopic graft experiments (Figures 4, 5, and 6).

At first, we examined whether the CC region exerts an attractive influence on cortical axons by placing E16.5 lateral CC explants, comprising the two neuronal populations of interest, adjacent to explants of medial (cingulate or frontal) cortex (Figure 4A_i_–4A_ii_). At E16.5, after 2 d in vitro, outgrowth in the quadrant closest to the CC aggregate was increased for axons originating from the cingulate and the frontal cortical area compared to that in the quadrant furthest away from the aggregate, indicating chemoattraction (Figure 4A_i_–4A_ii_ and 4C). By contrast, the septal and the IG regions were found to exert a repulsive action on cortical axons (Figure 4B_i_–4B_ii_, 4C, and unpublished data). At E16.5, after the cerebral hemispheres have fused, it was possible to ascertain the callosal identity of the axons, by using CC organotypic slices. DiI-labeled axons growing in E16.5 slice preparations from GAD67-GFP slices (unpublished data, *n* = 17 out of 22) or in E16.5 WT slices grafted with a CC from a GAD67-GFP embryo (Figure 4D_i_–4D_iii_; *n* = 4 out of 4) navigated across the midline as they normally do in vivo. In contrast, when small explants of E16.5 GAD67-GFP–positive lateral CC containing both neuronal populations were inserted into E16.5 heterotopic septal region of host slices, some DiI-labeled callosal axons were deflected from their normal trajectory, penetrated the SEP and innervated the transplants (Figure 4E_i_–4E_iii_, arrowheads; *n* = 7 out of 8). The enrichment of both types of CC neurons within small E16.5 lateral CC explants was confirmed by using GAD67-GFP–positive explants (Figure 4A_ii_ and Figure 4D_i_–4D_iii_ to 4F_i_–4F_iii_) and CR immunohistochemistry (Figure 4F_i_–4F_iii_), whereas the lack of astroglial cells was demonstrated by GFAP immunohistochemistry (unpublished data). Thus, these observations reveal the existence of an attractive activity for callosal axons located in the neuron-rich region of the CC.

We next determined the respective contribution of GAD67-GFP–positive GABAergic interneurons and CR-positive glutamatergic neurons of the CC to this guidance activity. To directly test the involvement of CC GABAergic neurons, we grafted in the SEP, explants of E14.5 or E16.5 GAD67-GFP medial ganglionic eminence (MGE) (Figure 5A_i_–5A_iii_), which generate the GABAergic interneurons of the CC (unpublished data). Interestingly, numerous axons left the callosal track, penetrated the repulsive SEP, and grew through migrating GAD67-GFP–positive interneurons originating from the MGE transplant (Figure 5A_i_–5A_iii_; *n* = 12 out of 15 for E14.5 MGE, and *n* = 17 out of 22 for E16.5 MGE). This attraction was specific for MGE-derived interneurons, since control explants of the lateral ganglionic eminence (LGE) did not attract callosal axons (Figure 5B_i_–5B_iii_; *n* = 5 out of 6). These observations strongly support the idea that CC GABAergic neurons directly contribute to the attraction of callosal axons. To estimate whether CC GABAergic neurons are the sole contributors of this guidance activity, we compared in coexplant experiments the quantity of cortical axons that were attracted by WT or *Mash1^−/−^* E16.5 lateral CC explants (Figure 5C_i_–5C_ii_, 5D_i_–5D_ii_, and 5E). *Mash1^−/−^* explants of the lateral CC, that contained glutamatergic neurons but are devoid of GABAergic interneurons (Figure S5D_i_ and S5F_i_), were found to exert a reduced chemoattraction on cortical axons compared to WT lateral CC explants that contain both neuronal populations (Figure 5E; −40%, *p*<0.05). The equal number of CR-positive neurons within small lateral CC explants of *Mash1^−/−^* compared to WT was confirmed by using CR immunohistochemistry (unpublished data). These results show that CC GABAergic interneurons contribute to part of the attractive activity of the CC on cortical axons.

To further test whether CR-positive glutamatergic neurons can also directly attract callosal axons, we took advantage of the fact that the E14.5 developing CC comprises CR-positive glutamatergic neurons and lacks GAD67-GFP–expressing GABAergic interneurons (Figure 1C_i_–1C_ii_). Coexplant experiments performed at E14.5 showed that cortical axons from the CCi were attracted by CC explants comprising only CR-positive neurons (Figure 6A_i_–6A_ii_ and 6B). In addition, heterochronic transplantation of E14.5 developing CC into the SEP of a E16.5 WT slice revealed that regions enriched in CR-positive neurons (Figure 6C_i_–6C_iii_) provided an attractive environment for callosal axons (Figure 6D_i_–6D_iii_, arrowheads; *n* = 11 out of 13).

Altogether, coexplant and transplantation experiments indicate that CC neuronal populations exert an attracting influence on callosal axons, which is mediated by both GABAergic and glutamatergic CR-positive neurons.

### Sema3C Is Expressed by CR-Positive Glutamatergic Neurons and Contributes to the Attractive Activity on Callosal Axons

**Figure 7:**
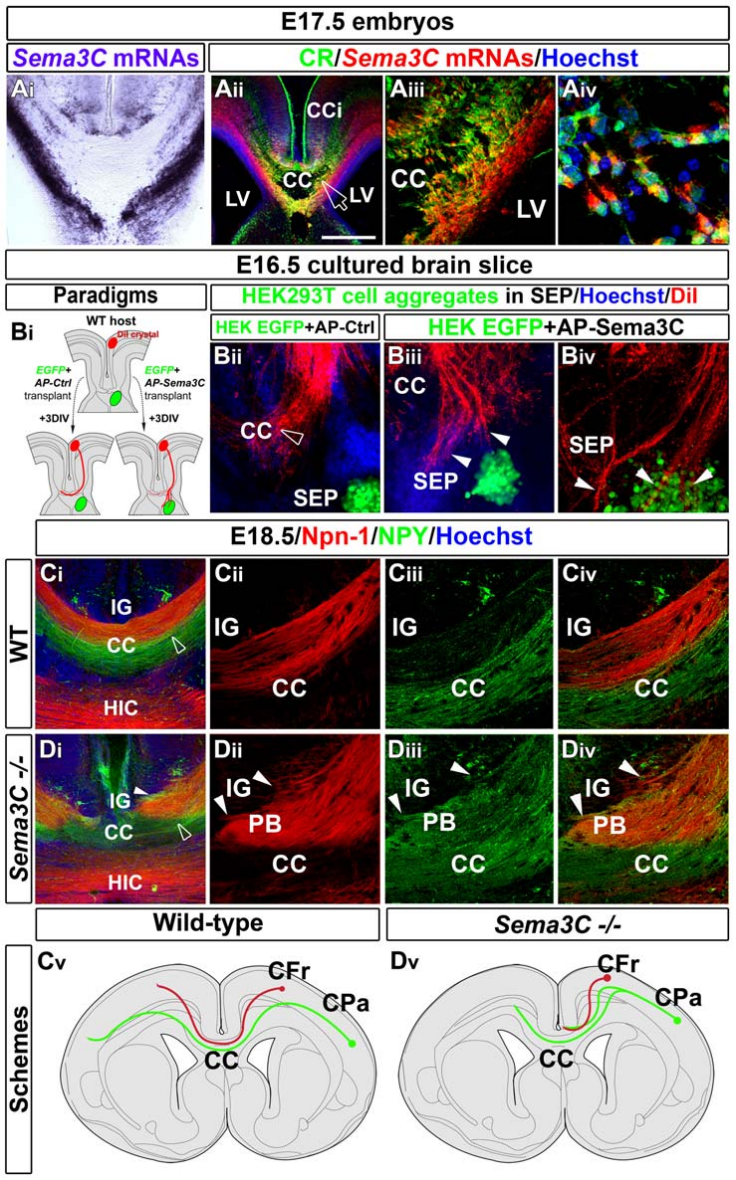
Sema3C expressed by CR-positive glutamatergic CC neurons control callosal axons navigation. (A) In situ hybridization for Sema3C combined (A_ii_–A_iv_) or not (A_i_) with immunohistochemical staining for CR in coronal telencephalon sections at E17.5. (A_iii_–A_iv_) are high-power views of the CC seen in (A_ii_) (open arrow). CR-positive glutamatergic neurons of the CC are seen to express strong levels of *Sema3C* mRNAs. (B_i_) Experimental paradigm used to test the effect of AP-control (B_ii_) or AP-Sema3C (B_iii_–B_iv_) transfected HEK cell aggregates on the growth of callosal axons. (B_ii_) DiI-positive callosal axons navigate normally (open arrowhead) when they are confronted with control HEK cells. (B_iii_–B_iv_) DiI staining showing that callosal axons are misrouted and attracted by GFP^+^ aggregates of HEK cells secreting Sema3C (arrowheads). (C and D) E18.5 CC coronal sections showing callosal axon paths of WT (C_i_–C_iv_) and *Sema3C^−/−^* (D_i_–D_iv_) mice. C_ii_–C_iv_ and D_ii_–D_iv_ are high-power views of the CC seen in (C_i_ and D_i_), respectively (open arrowheads). Whereas callosal axons of the dorsal path (Npn-1–labeled) and of the ventral path (NPY-labeled) are segregated in the WT CC, they intermix in the mutant and form ectopic axonal Probst bundles (PB) that invade the indusium griseum (IG) (arrowheads in [D_ii_–D_iv_]). Schematic drawing of callosal fiber trajectories in the WT (C_v_) and *Sema3C^−/−^* (D_v_) brains, respectively. Bar indicates 435 µm in (A_ii_, C_i_, and D_i_), 180 µm in (A_i_), 220 µm in (B_ii_, B_iii_, and B_iv_), 110 µm in (A_iii_, C_ii_, C_iii_, C_iv_, D_ii_, D_iii_, and D_iv_), and 45 µm in (A_iv_). HIC, hippocampal commissure; LV, lateral ventricle.

In search for candidate molecular signals mediating the attractive activity on callosal axons, we found that *Sema3C* is strongly expressed only in the subcortical white matter and especially the CC region (Figure 7A_i_ and Figure S6I_i_–S6I_ii_), as previously observed [26]. In the CC, colabeling experiments revealed that *Sema3C* mRNA expression is restricted to CR-positive glutamatergic neurons (Figure 7A_ii_–7A_iv_). The *Sema3C* mRNAs were never detected in GAD67-GFP–positive interneurons (Figure S8A_i_–S8A_ii_) or GFAP-positive astroglial cells (Figure S8B_i_–S8B_ii_). Since Sema3C has been described to act as an attractive factor for neocortical and cingulate axons in vitro [26],[40],[41], CR-positive glutamatergic neurons of the CC might exert their attractive effect on callosal axons through the action of Sema3C.

To test this possibility, aggregates of Sema3C-expressing HEK293T cells were placed in the repulsive septal region of E16.5 WT slices (Figure 7B_i_). Callosal axons were misrouted from their normal path and invaded cell aggregates expressing Sema3C (Figure 7B_iii_–7B_iv_; arrowheads; *n* = 13 out of 16), whereas control cell aggregates did not affect the growth of callosal axons (Figure 7B_ii_; open arrowheads; *n* = 7 out of 8). Thus, localized expression of Sema3C in slice cultures directs callosal axon outgrowth. In addition, experiments made with explants of E14.5 and E16.5 cingulate or frontal cortices and aggregates of Sema3C-expressing HEK293T cells indicate that pioneer cortical axons and later-growing callosal axons are chemo-attracted by Sema3C as early as E14.5 (unpublished data).

To determine the in vivo function of Sema3C in the developing CC, we examined the brains of mutant mice inactivated for the *Sema3C* gene (Figure 7D_i_–7D_v_ and Figure S8D_i_–S8D_ii_). CR and GFAP immunohistochemistry at E16.5 and E18.5 indicated that the position and organization of the CR-positive glutamatergic neurons and glial cell populations within the CC is indistinguishable in WT and *Sema3C^−/−^* mice, suggesting that their development is not sensitive to the loss of *Sema3C* (compare Figure S8C_i_ with S8D_i_ and Figure S8C_ii_ with Figure S8D_ii_, respectively). *Sema3C^−/−^* mice exhibited partial to severe AgCC. When the agenesis was partial, all dorsal Npn-1–positive axons failed to cross the midline, whereas part of ventral callosal axons labeled for NPY were able to cross (compare Figure 7C_i_–7C_v_ with Figure 7D_i_–7D_v_). Misguided callosal axons formed Probst bundles within the IG (Figure 7D_i_–7D_iv_, arrowheads). In some cases, *Sema3C^−/−^* mice displayed severe AgCC characterized by midline fusion defects and a complete failure of any callosal axons to cross the midline at the level of the CC main body (compare Figure S8C_i_–S8C_ii_ with Figure S8D_i_–S8D_ii_).

Taken together, these results reveal that guidance mechanisms of callosal axons rely in part on Sema3C, which contributes to the chemoattractive effect of CR-positive glutamatergic neurons on callosal axons.

### Sema3C Directs Cortical Axon Growth via Npn-1

**Figure 8:**
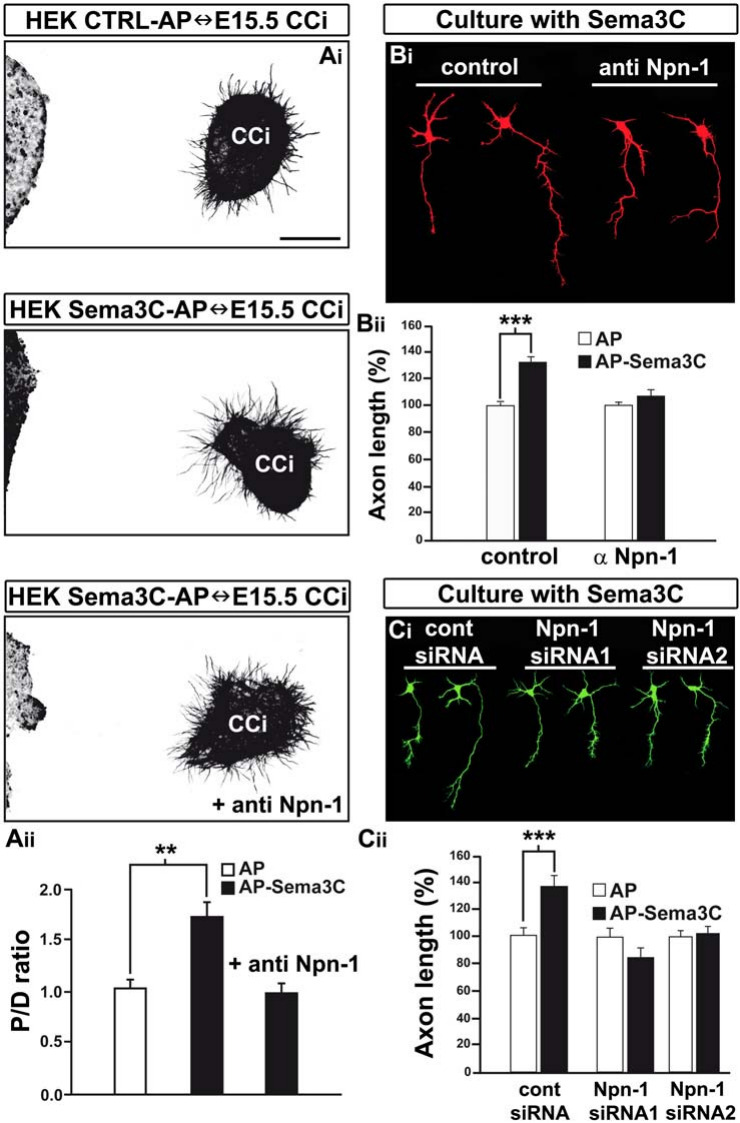
Sema3C acts through Npn-1 to attract and promote growth of cortical axons. (A–C) Typical patterns of axonal outgrowth (A_i_) from explants of E15.5 CCi cocultured with AP-control and AP-Sema3C–expressing HEK293T cells in the presence or absence of 20 µg/ml polyclonal anti–Npn-1 antibodies. (A_ii_) Quantification of the axonal guidance responses to AP-control and AP-Sema3C–expressing HEK293T cells. Data are expressed as a *P/D* ratio, where *P* and *D* are the mean lengths of axons in the quadrants proximal and distal to the cell aggregate [77]. In control conditions, a *P/D* ratio close to 1 (*P/D* ratio = 1.04±0.09) indicates radial outgrowth. AP-Sema3C causes strong chemoattraction of cortical axons (*P/D* ratio = 1.73±0.15). This effect is blocked by anti–Npn-1 antibodies (*P/D* ratio = 0.99±0.09). (B_i_) Typical images of dissociated neurons from E15.5 CCi cultured in the presence or absence of 10 µg/ml polyclonal anti–Npn-1, and of 10 nM AP-Sema3C. (B_ii_) Histograms of Sema3C effects on the growth of dissociated neurons from CCi. Data are presented as mean axonal length ±SEM and are normalized to 100% with respect to values obtained in control conditions. The anti–Npn-1 antibodies prevent stimulation of axon growth by Sema3C. (C_i_) Dissociated neurons of CCi electroporated with GFP-expressing vector together with either control siRNA or Npn-1 siRNAs 1 and 2 (see Materials and Methods) were cultured in the presence or absence of 10 nM AP-Sema3C. (C_ii_) Quantification of the effects of Npn-1 siRNAs on axon length. Knock-down of Npn-1 completely abolishes the growth-promoting effect of Sema3C. Data are mean axonal lengths ±SEM calculated as a percentage of mean values obtained in control conditions for each experiment. Double asterisks (**) indicate significantly different with *p*<0.01; triple asterisks (***) indicate significantly different with *p*<0.001.

The precise identity of the endogenous neuronal receptor for Sema3C remains unclear. In vitro, Sema3C binds with high affinity to both Npn-1 and its close homolog Npn-2 [42]. Since Npn-1, but not Npn-2, is expressed on callosal axons (see Figure 2A_i_–2A_ii_, 2B_i_–2B_ii_, 2D_i_–2D_ii_, Figure 3C_i_–3C_ii_, and 7C_i_–7C_iv_; and unpublished data) and Semaphorin/Npn-1 signaling is critical for CC development [26],[29], we examined whether Npn-1 was necessary to allow callosal axons to respond to Sema3C. We placed aggregates of Sema3C-expressing HEK293T cells adjacent to explants of E15.5 medial cortex (Figure 8A_i_–8A_ii_). After 2 d in vitro, axonal growth in the quadrant closest to the aggregate was increased by 70% at E15.5 compared to that in the quadrant farthest away from the aggregate (*p*<0.01; Figure 8A_i_–8A_ii_), indicating chemoattraction. Consistently, adding recombinant Sema3C (5 to 10 nM) to dissociated neurons from medial cortex increased axon length by 35% compared to the control condition (*p*<0.001; Figure 8B_i_–8B_ii_). Npn-1 blocking antibodies abolished both the attractive and growth-promoting responses of cortical neurons to Sema3C (Figure 8A_i_–8A_ii_ and 8B_i_–8B_ii_) and disturbed DiI-labeled callosal axons navigation in E16.5 brain slices (unpublished data, *n* = 14 out of 19). To exclude the possibility of nonspecific antibody binding, we knocked down endogenous Npn-1 in dissociated cortical neurons using two different small interfering RNA (siRNA) sequences that efficiently silenced expression of Npn-1 without affecting Npn-2 levels, as assessed by antibody staining (unpublished data). Remarkably, both siRNAs completely abrogated the positive effect of Sema3C on axon growth (Figure 8C_i_–8C_ii_). Taken together, these results strongly suggest that Npn-1 is necessary for mediating the attractive response of callosal axons to Sema3C.

## Discussion

In this study, we show that two transient neuronal subpopulations, one CR/glutamatergic and the other Mash1/GABAergic, occupy a strategic position for contributing to the guidance of nascent callosal axons. *Mash1^−/−^* mice that lack GABAergic neurons possess major pathfinding defects in the CC, and grafting WT CC comprising midline neurons in a *Mash1^−/−^* slice rescued this phenotype. In addition, we show that both neuronal populations possess the ability to chemoattract callosal axons and that this activity is dependent in part on Sema3C function in the CR-positive glutamatergic population. Consistent with this observation, Sema3C is required in vivo for the proper development of the CC pathway. Taken together, our work provides strong evidence for a role in callosal axon guidance by the two transient populations we characterized in this study. Notably, these activities appear distinct from those emanating from glial guidepost cells. As such, our findings show that two previously uncharacterized neuronal populations converge towards the midline and form a cellular network that is essential for controlling callosal axon navigation (model in Figure 2G_i_–2G_ii_).

### Roles for CC Neurons in the Guidance of Callosal Axons

We have revealed the existence of two populations of glutamatergic and GABAergic neurons that although arising from distinct sources, converge on the interhemispheric fissure prior to the arrival of CC axons. The precise origins of these two CC neuronal populations have yet to be determined. Our observations suggest that CR-positive glutamatergic neurons invade the CC through a tangential subpial migration and may thus correspond to cortical pioneer neurons that originate from the retrobulbar ventricle [43],[44]. In contrast, our fate-mapping and tracing experiments indicate that the GABAergic interneurons of the CC originate in the MGE (unpublished data) as described for a majority of cortical interneurons in mice [45]–[51].

A few studies have reported the presence of neurons within or around the CC, such as CR-positive neurons in the mouse and human “glial” sling [16],[17] and scattered neurons in the cat CC during early postnatal life [18],[19]. It was proposed that these neurons were migrating through the CC [19] or below the CC in the sling [10]. Our study provides an evaluation of the positioning, development, and character of these populations and demonstrates that their presence within the CC is transient.

Moreover, our data strongly support a requirement for these neurons in the guidance of callosal axons. First, we identified a close structural association between the neurons of the CC and the callosal axons during embryonic development. The intimate relationship between these neurons and the incoming callosal afferents is further bolstered by our 3-D analysis showing that CR-positive glutamatergic neurons form a complex multicellular network with the transient GABAergic interneuron population we identified. The integrity of the CC multicellular network formed by GABAergic and glutamatergic neurons is required for normal CC axonal navigation, as shown by both our analysis of *Mash1* mutant mice and our grafting experiments. Our present work reveals that the guidance of callosal axons is actively mediated through the chemotropic actions of the two novel neuronal populations of the CC that we examined. Moreover, our study demonstrates that CR-positive glutamatergic neurons exert a direct attractive influence on callosal axons via *Sema3C* expression.

This function of the CC neurons fits well with the emerging notion that migrating neurons may have a role in axon pathfinding. It has recently been found that thalamocortical axon growth relies on the early tangential migration of a population of GABAergic neurons within the ventral telencephalon [52]. In addition, the lateral olfactory tract (LOT) projections are guided by early generated neurons, named “LOT neurons,” that migrate tangentially [53],[54]. Similar functions have been reported for CD44-positive neurons in the guidance of retinal axons at the optic chiasm [55] and Cajal-Retzius reelin/calretinin-positive neurons in the establishment of hippocampal projections [56].

### CC Neurons Participate in Callosal Axon Guidance via Sema3C/Npn-1 Signaling

Our work demonstrates that a neuronal-rich region of the CC attracts callosal axons, at least in part through the expression by glutamatergic CC neurons of the guidance cue Sema3C. Initially, the *Sema3C* gene is strongly expressed by guidepost CR-positive glutamatergic neurons adjacent to the midline prior to the entrance of callosal axons. In organotypic slices, Sema3C-expressing HEK293T cells attract callosal axons into heterotopic regions. These observations, using a specific guidance assay for CC axons, extend previous in vitro studies showing that Sema3C acts as an attractive guidance signal for neocortical and cingulate axons [26],[40],[41]. It was known that the Sema3C repulsive activity is mediated via Npn-1/Npn-2 heterodimers or Npn-2/Npn-2 homodimers [57], but the nature of the receptor mediating the attractive effect was not yet characterized. Here, we show that inhibiting selectively the Npn-1 receptor abolished completely the attractive and outgrowth-promoting effects of Sema3C. Moreover, callosal axons are found to express Npn-1, but not Npn-2. Therefore, our results reveal that Npn-1 can serve as a Sema3C receptor to mediate chemoattraction. Neuropilins require a signaling coreceptor to mediate semaphorin function. For example, PlexinAs and L1-CAM are responsible for transducing Sema3A repulsive response via Npn-1 in cortical neurons [58],[59]. Other transmembrane proteins, including the tyrosine kinase receptors Met, ERBB2, OTK, and VEGFR2, participate in semaphorin responses by regulating diverse intracellular signaling events and functional outcomes [60]–[62]. The transducer that mediates Sema3C attractive response remains so far undefined. It will be important to analyze whether the assembly of specific subunits combinations confers unique ligand-binding properties of semaphorin receptors, and whether different Npn-1 receptor complexes coexist on cortical axons, dictating either Sema3A-mediated repulsion or Sema3C-mediated attraction.

Although it has long been recognized that Sema3C regulates the formation of the cardiovascular system [63], its in vivo function in the central nervous system remains relatively unexplored. Here, we observed that the development of the CC path depends on Sema3C expression. Indeed, callosal axons fail to grow or navigate correctly through the CC of mutant mice lacking *Sema3C* gene function. These results shed new light on previous studies showing that mice in which Npn-1 is unable to bind Semas exhibit CC axonal pathfinding defects [26],[29]. The similarity between the *Npn-1^Sema−^* mice and *Sema3C^−/−^* mice suggests that Sema3C is the ligand required for directing Npn-1–mediated callosal axon navigation. Taken together, these results reveal that CR-positive glutamatergic neurons within the dorsal midline territory control callosal axon navigation, at least in part, through a Sema3C-dependent mechanism. Therefore, transient CC guidepost neurons play a central role in mediating the guidance cues required for callosal axon pathway formation.

### Neurons and Glia Cooperate to Guide Callosal Axons

Previous studies on CC development emphasized the role of glial cells. In Silver and Ogawa's study [11], aberrant callosal axons maintained a potential to regrow upon the surface of a glia-covered scaffold after mouse embryos were made surgically acallosal at E16.5. Other studies indicate that astroglial cells of the GW and IG direct callosal pathfinding at the midline by secreting guidance cues [7],[8]. Our work demonstrates that, in addition, CC formation requires the presence of specific neuronal populations. What is the relative contribution of neurons and glia to CC formation? Preliminary results indicate that neurons and astroglial cells of the CC intermingle to form a complex 3-D structure and that glutamatergic CR-positive neurons lie along the radial glial processes. Therefore, in addition to secreting guidance factors, glial cell populations may aid in the establishment of the CC neurons through more complex trophic or signaling interactions. Indeed, the interplay between the neuronal and glial cells within the midline will be intriguing to investigate in the future.

## Materials and Methods

### Animals

All animal research has been conducted according to relevant national and international guidelines. WT mice maintained in a C57Bl/6 genetic background were used for developmental analysis of the CC. We used heterozygous GAD67^_^GFP (Δneo) mice [33], which will be referred to as GAD67-GFP mice in this work. Experimental animals were obtained by mating C57Bl/6 mice with heterozygous GAD67^_^GFP mice. GAD67^_^GFP embryos can be recognized by their GFP fluorescence. NINDS GENSAT BAC Transgenic mice for *Ascl1* (Ascl1-EGFP)1Gsat/Mmnc (MMRC) referred to as Mash1-GFP in this work were maintained in a C57Bl/6 background and were recognized by their GFP fluorescence. *Mash1* KO heterozygous mice were maintained in a mixed C57Bl/6 and DBA background and crossed to produce homozygous embryos [64]. *Mash1* heterozygous mice were also crossed with a transgenic mouse line expressing GFP ubiquitously [65] in order to produce GFP-positive *Mash1^−/−^* embryos. PCR genotyping of these lines was performed as described previously [52]. Heterozygous embryos did not show any phenotype and were used as controls. *Sema3C* heterozygous mice were maintained in a CD1 background and mated to obtain *Sema3C^−/−^* embryos. The genotype of the offspring was determined by PCR as described [63]. For staging of embryos, midday of the day of vaginal plug formation was considered as embryonic day 0.5 (E0.5).

Embryos were collected by Caesarean section and killed by decapitation. Their brains were dissected and fixed by immersion overnight at 4°C in a solution containing 4% paraformaldehyde (PFA) in 0.1 M phosphate buffer (pH 7.4). Postnatal mice were deeply anaesthetized and perfused with the same fixative, and their brains postfixed 4 h. Brains were cryoprotected in 30% sucrose, and cut in coronal 50-µm-thick frozen sections for staining.

### Production of AP-Sema3C

AP-Sema3C was obtained by cloning cDNA encoding mouse Sema3C in pAPtag-5 vector (GenHunter Corporation), which contains a sequence coding for secreted alkaline phosphatase. To produce AP-tagged proteins, HEK293T cells were transfected with the AP-Sema3C vector or empty pAPtag-5 vector as control, using lipofectamin plus (Invitrogen) or fugen (Roche). After 3 d of culture in Opti-MEM serum-free medium, the supernatant was collected and concentrated using Centricon filters (Millipore). AP activity was assessed as described [29].

### Slice Culture Experiments

We developed an in vitro model of CC organotypic slices adapted from a previously published telencephalic slice culture preparation [45],[52],[66]–[68] and CC preparation [7]. Embryos were placed in ice cold dissecting medium (MEM Gibco ref 11012-044 with 15 mM glucose and 10 mM Tris [pH 7–9]). Brains were removed and embedded in 3% low-melting point agarose (Invitrogen); 250-µm-thick coronal sections were then cut using a vibratome filled with cold dissecting medium, and slices at the level of the CC were collected in the same medium. CC slices were cultured on nuclepore Track-Etch membrane (1-µm pore size; Whatman) or PET cell inserts (1-µm pore size; Beckton-Dickinson) in tissue dishes containing 1 ml of BME/HBSS (Invitrogen) supplemented with glutamine, 5% horse serum, and Pen/Strep [52].

For CC transplantation experimentation, slices from E16.5 embryos were selected since at this early stage of development, the CC contained its whole complement of guide post cells and only the pioneer CC axons of ventral cingulate origin [36],[37]. It is critical that cultured hemispheres are already joined for the differentiation of the CC in vitro. In our slice assay, as in vivo, the callosal axons from dorsolateral neocortex develop later, and after E16.5, their growth cones enter the CC region in successive streams over a period of several days. Our slice assay performed at E16.5 allowed us to study: (1) the function of both CC guidepost neuronal populations that have reached the CC midline at that stage, (2) the outgrowth properties of the majority of callosal axons that are growing through the CC after E16.5, and (3) the effects of transplantations and pharmacological (guidance factors, lesions) manipulation on callosal axons navigations.

To define the putative function of CC neurons in attracting callosal axons, the transplantation assay was performed at E16.5 to analyze the navigation of WT early callosal axons labeled for DiI after insertion of small DiI crystals into the frontal cortex of slices. Small explants of E14.5 corticoseptal boundary comprising only CR-positive glutamatergic neurons or E16.5 lateral CC IZ comprising both neuronal populations were excised using tungsten needles and transplanted into the SEP of E16.5 host slices. After incubation for 48–64 h, the slices were fixed, and axon trajectories through the various regions were analyzed by confocal analysis. In most of our transplantation experiments of CC (>90%), we observed that axons grew without any difficulty through small or large transplants, and only cases with axons penetrating into the grafted explants were counted as positive results for attraction.

We found that CC GABAergic interneurons are generated by the medial ganglionic eminence (MGE) from E14.5 to E16.5 (unpublished data). To define the putative function of the CC GABAergic interneurons we transplanted small explants of E14.5 or E16.5 MGE into the SEP of an E16.5 slice as described above. As a control, we used small explants of E16.5 lateral ganglionic eminence (LGE) that do not generate CC GABAergic interneurons. In this assay, cases with axons growing along GAD67-GFP^+^ interneurons originating from the grafted explants were counted as positive results for attraction.

For the Sema3C study, HEK293T cells were transfected with an AP-control plasmid or an AP-Sema3C plasmid (see above). To highlight HEK293T transfected cells, a pEGFP plasmid was coexpressed. Aggregates of HEK293T transfected cells prepared by high-density culture within an inverted drop of medium were transplanted into the CCi, CC, or SEP of host slices as described before [68]. For the Npn-1 study, the Npn-1–blocking antibody (R&D systems) was added at the final concentration of 5 µg/ml.

For the *Mash1* study, the transplantation assay was performed at E16.5 to analyze the growth of WT (*Mash1^+/+^*; *Mash1^+/−^*) or *Mash1^−/−^* GFP-positive callosal axons within CC of WT (*Mash1^+/+^*; *Mash1^+/−^*) or *Mash1^−/−^* slices. Since heterozygous embryos did not show any phenotype, they were also used as controls. Portions of the frontal cortex with underlying white matter and CC from donor slices were excised using tungsten needles and transplanted into host slices from which the equivalent region had been removed. After incubation for 48–64 h, the slices were fixed and immunostained for GFP before confocal analysis.

### Coculture and Dissociated Neuronal Cultures

Cocultures were performed as described [52],[69]–[71]. Explants of E14.5, E16.5 CCi, explants of E16.5 frontal cortices, explants of E14.5, E16.5 SEP, or explants of E16.5 IG were cocultured with CC explants of the corresponding ages. Explants of E14.5 and E15.5 CCi, or E14.5, E15, and E16.5 frontal cortex were cocultured with HEK293T cell aggregates secreting AP-Sema3C or control AP. For the *Mash1* study, the coexplant assay was performed at E16.5 to analyze the growth of WT (*Mash1^+/+^*; *Mash1^+/−^*) or *Mash1^−/−^* cortical axons confronted with WT (*Mash1^+/+^*; *Mash1^+/−^*) or *Mash1^−/−^* CC.

For dissociated cell cultures, neurons were dissociated and plated onto polylysine/laminin-coated four-well plates (Nunc) in Neurobasal medium supplemented with 1 mM glutamine, 1∶50 B27 (GIBCO), and AP control or AP-Sema3C supernatants (see above). In some experiments, neurons were cultured in the presence of anti–Npn-1 (R&D Systems). Efficient knock-down of Npn-1 was obtained using the following siRNA sequences: 5′-AAUCAGAGUUCCCGACAUAUU-3′ (Npn-1 siRNA1) and 5′-UGUCAAGACUUACAGAGUAUU-3′ (Npn-1 siRNA2). Neurons were coelectroporated with a pCAGGS-GFP vector and with different siRNAs (100 pmol) as described [72]. Quantification of axonal growth and guidance was performed as described before [73], or by using a measuring program built in MatLab software that allows to compare the density of immunolabeled axons in the proximal region facing the source of guidance cues and the distal region.

### In Situ Hybridization

Sema3C plasmid was linearized with EcoRI (New England Biolabs) for antisense RNA synthesis by T7 polymerase (Promega) and with XhoI (New England Biolabs) for sense RNA synthesis by T3 polymerase (Promega). EphA4, Npn-1, EphB1 plasmids were linearized with SacI (New England Biolabs) for antisense RNA synthesis by T3 polymerase (Promega). ephrinB2 plasmid was linearized with BamH1 (New England Biolabs) for antisense RNA synthesis by sp6 polymerase (Promega). Slit2 plasmid was linearized with Xba1 (New England Biolabs) for antisense RNA synthesis by T7 polymerase (Promega). For in situ hybridization, brains were dissected and fixed by immersion overnight at 4°C in a solution containing 4% paraformaldehyde (PFA) in PBS. Free-floating vibratome sections (100 µm) were hybridized with digoxigenin-labeled cRNA probe as described before [74]. To combine in situ hybridization and immunofluorescence, Fast Red (Roche) was used as an alkaline phosphatase fluorescent substrate.

### Immunocytochemistry

Monoclonal antibodies were human DCC receptor and NeuN (Chemicon); Nestin (Pharmingen); and SNAP25 (Stemberger Monoclonal). Rat monoclonal antibody was L1 (Chemicon). Rabbit polyclonal antibodies were calbindin and calretinin (Swant); GABA (Sigma); GFAP (DAKO); GFP (Molecular Probes); GLAST, Tbr1, and Tbr2 (Chemicon), Satb2 (gift from V. Tarabykin); Emx1 (gift from A. Trembleau); and cleaved caspase 3 (Cell Signaling). Goat polyclonal antibodies were calretinin (Swant); Npn-1 and Npn-2 (R&D System); and NPY (gift from W. W. Blessing, Flinders University, Melbourne, Australia). Guinea pig polyclonal antibodies were VGLUT1 and VGLUT2 (Chemicon). To label ephrin-A5 binding sites, we used the ephrinA5 chimera human Fc (R&D Systems).

#### Fluorescence immunostaining

Unspecific binding was blocked by adding 2% normal horse serum during preincubation and incubations in 1× PBS solutions containing 0.3% Triton X-100. The primary antibodies were detected with donkey or goat Cy3-, Cy2, Alexa 594, Alexa 488, and Oregon Green antibodies (Jackson ImmunoResearch and Molecular Probes). Sections were counterstained with Hoechst 33258 (Molecular Probes), mounted on glass slides, and covered in Mowiol 4-88 (Calbiochem). Coexplants were counterstained with Hoechst 33258 (Molecular Probes) and covered in Vectashield.

### Axonal Tracing

After overnight fixation in 4% PFA at 4°C, fine glass needles covered with the fluorescent carbocyanide dye DiI (1,1′-dioctadecyl 3,3,3′,3′-tetramethylindocarbocyanine perchlorate or DiA (4-[4-(dihexadecyl amino)styryl]*N*-methyl-pyridinium iodide (Molecular Probes) were placed in single or multiple locations in the neocortex [75]. After 4–8 wk at 37°C in 4% PFA or PBS to allow dye diffusion, the samples were embedded in 5% agarose and cut into 100-µm-thick sections on a vibratome. Counterstaining was with Hoechst (Molecular Probes).

### Imaging

Fluorescent-stained sections were imaged with confocal microscopes (Zeiss LSM 510 Meta or Leica SP5) equipped with 10×, 20×, 40× oil Plan-NEOFLUAR, and 63× oil, 100× oil Plan-Apochromat objectives. Fluorophore excitation and scanning were done with an Argon laser 458, 488, 514 nm (blue excitation for GFP, Alexa488, CY2, and DiA), with a HeNe1 laser 543 nm (green excitation for Alexa 594, CY3, and DiI) and a Diode laser 405 nm (for Hoechst staining). *Z*-stacks of 10–15 plans were acquired for each CC coronal section in a multitrack mode avoiding crosstalk artifacts of the fluorochromes. *Z*-stacks of 40–50 sections were acquired for each CC section for the creation of isosurfaces with Imaris4.3 software.

#### Imaris images processing

All 3-D *Z*-stack reconstructions and image processing were performed with Imaris 4.3 software (Bitplane). Some image stacks contained approximately 40 sections each, giving an excellent *z*-axis resolution. To create real 3-D datasets, we used the mode “Surpass” of Imaris. Single sections of a *Z*-stack were displayed by using the “Slice” mode of Imaris. The generation of isosurfaces (object defining a surface surrounding voxels located between two threshold values) allowed us to visualize the contours of cells and to delimit the cell-free spaces between the neurons of the CC. Using a navigator function of IMARIS 4.3 software in the “Animation mode,” it was then possible to explore the organization of the cell-free spaces within the thickness of the CC. The Animation mode allows Key-Frame Animation of the exploration of CC slices to be saved in AVI file format. The colocalization between two fluorochromes was calculated and visualized by creating a yellow channel using Imaris. Figures were processed in AdobePhotoshop CS2, and schematic illustrations were produced using Adobe Illustrator CS2.

### Ultrastructure

E16.5 and E18.5 embryos were killed by decapitation. Brains were dissected and fixed by immersion for 24 h at 4°C in a solution containing 2% glutaraldehyde and 4% paraformaldehyde in 0.1 M phosphate buffer (pH 7.4) with the addition of 2% sucrose. The brains were then rinsed in 0.1 M cacodylate buffer (pH 7.4), postfixed at room temperature for 2 h in 1% OsO_4_, dehydrated in graded ethanols, and embedded in Epon. The regions containing the CC of the embedded brains were trimmed and mounted on blocks to cut semithin and ultrathin sections. The ultrathin sections were mounted on Formvar-coated single-slot grids and contrasted with 2% uranyl acetate and 0.2% lead citrate.

For pre-embedding immunocytochemistry, embryonic brains were fixed by immersion in a solution containing 4% paraformaldehyde and 0.1% glutaraldehyde in 0.1 M phosphate buffer (pH 7.4) supplemented with 2% sucrose for 24 h. Fifty-micrometer-thick sections were cut with a vibratome and immunoreacted. Endogenous peroxidase reaction was quenched with 0.5% hydrogen peroxide in methanol, and unspecific binding was blocked by adding 2% normal horse serum during preincubation and incubations in Tris-buffered solutions. The primary antibodies were detected with biotinylated secondary antibodies (Jackson ImmunoResearch) and the Vector-Elite ABC kit (Vector Laboratories). Following the diaminobenzidene reaction, sections were dehydrated and embedded in Epon. The plastic-embedded specimens were prepared for ultrathin sectioning following the same protocol as above.

### CC Cell Populations Analysis

In slices of WT mice, the CR^+^, Tbr1^+^, and GAD67-GFP^+^ neurons in the CC were counted at E16.5 and E18.5 as the number of cells in the CC region from at least five slices per condition. To study the total neuron number through the CC, the values were reported as a percentage of the total number of the cells encountered within the same region of the CC and labeled by Hoechst staining. To study the neuronal subpopulations repartition, the values were reported as a percentage of the total number of labeled neurons encountered within the same region of the CC.

In slices of WT and *Mash1^−/−^* mice, the GABAergic interneurons in CC were counted as the number of neurons labeled for GABA per surface unit from at least five slices per condition.

### Statistical Analysis

For all analyses, values from at least three separate experiments were at first tested for normality. Values that followed a normal distribution were compared using Student *t*-test or one-way ANOVA and Fisher *t*-tests. Values that did not follow a normal distribution were compared using Mann-Whitney and Kolmogorov-Smirnov nonparametric tests.

### Atlas and Nomenclature

The nomenclature for callosal development is based on the *Atlas of the Prenatal Mouse Brain* [76]. On the basis of our results, we considered that the CC is divided into two sectors: the medial part is bordered dorsally by the IG and the longitudinal fissure, and ventrally by the GW and the dorsal limit of the septal area. The lateral part comprises the white matter bordered by the CCi superficially, and by the ventricular zone between the GW and the mediodorsal angle of the lateral ventricle towards the ventricular side.

## Supporting Information

Figure S1 **CC neuronal subpopulations and complementary organization of CC neurons and astroglial cells during embryonic development.** (A_i_) Bars (means±standard error of the mean [SEM] from a sample of 1,415 CR^+^ neurons at E16.5 and 252 CR^+^ neurons at E18.5) represent the percentage of CC CR^+^ neurons expressing Tbr1. (A_ii_ and A_iii_) Repartition of Tbr1^+^ and CR^+^ neuronal populations of the CC IZ at E16.5 (A_ii_) and E18.5 (A_iii_). Bars (means±SEM from a sample of 2,108 neurons in [A_ii_] and 312 in [A_iii_]) represent the percentage of neurons expressing or not Tbr1 and CR compared to the total number of neurons of the CC labeled with Tbr1 and/or CR. (B_i_) Bars (means±SEM from a sample of 1,907 CR^+^ neurons at E16.5 and 1,898 CR^+^ neurons at E18.5) represent the percentage of CC CR^+^ neurons expressing the GAD67-GFP. (B_ii_ and B_iii_) Repartition of GAD67-GFP^+^ and CR^+^ neuronal populations of the CC IZ at E16.5 (B_ii_) and E18.5 (B_iii_). Bars (means±SEM from a sample of 2,580 neurons in [B_ii_] and 3,442 in [B_iii_]) represent the percentage of neurons expressing or not the GAD67-GFP and the CR compared to the total number of neurons of the CC labeled with GAD67-GFP and/or CR. (C and D) Immunohistochemical staining for GFAP (C_i_–C_ii_) and nestin (D_i_–D_ii_) in coronal telencephalon sections from mice expressing Mash1-GFP at E18.5. (C_ii_ and D_ii_) are higher power views of the portion of the CC seen in (C_i_ and D_i_) (open arrowheads), respectively. (D_iii_–D_iv_) Illustration of the isosurfaces obtained from the Mash1-GFP and nestin staining in (D_ii_). Mash1-GFP GABAergic interneurons (arrowheads) migrate tangentially within the CC through radial glial processes that extend from the ventricular zone to the cortical marginal zone. (E) Double immunohistochemistry for CR and nestin (E_i_–E_iv_) in coronal sections from E18.5 mice. (E_ii_–E_iv_) are higher power views of the CC seen in (E_i_) (open arrowhead) and (E_v_–E_vi_) are isosurface illustrations obtained from the CR and nestin staining in E_iii_. CR-positive neurons (arrowheads) of the CC are positioned along nestin-positive radial processes. Bar indicates 220 µm in (C_i_, D_i_, and E_i_), 110 µm in (C_ii_ and E_ii_), and 70 µm in (D_ii_, E_iii_, and E_iv_). HIC, hippocampal commissure; MZG, midline zipper glia.
(8.72 MB TIF)

Figure S2 **Callosal axons exhibit a dorsoventral topographic organization within the CC.** (A) Coronal sections from E15.5 mice showing callosal axons labeled by insertion of DiI and DiA crystals, respectively, in the frontal (CFr) and parietal (CPa) cortices. Higher magnifications of the CC (A_ii_) and of the lateral white matter (A_iv_) are illustrated, respectively, in (A_i_ and A_iii_) (open arrowheads). Arrowheads in (A_ii_ and A_iv_) point to callosal axon endings. Callosal axons from the frontal cortex have not yet reached the future CC region at E15.5, whereas the callosal axons from the parietal cortex are growing through the white matter bellow the frontal cortex. (B and C) Coronal sections from E16.5 mice showing callosal axons labeled by insertion of DiI in the cingulate (CCi) (B_i_–B_ii_) and frontal (C_i_–C_ii_) cortices. Higher magnifications of the CC (B_ii_ and C_ii_) are illustrated, respectively, in (B_i_ and C_i_) (open arrowheads). Arrowheads in (B_ii_ and C_ii_) point to callosal axon endings. At E16.5, although pioneer callosal axons from the CCi have already crossed the CC midline, the callosal axons from the frontal cortex have not yet reached the CC midline. (D_i_–D_iv_) Coronal sections from P3 mice showing callosal axons labeled by insertion of DiA and DiI crystals, respectively, in the frontal cortex (CFr) and the parietal cortex (CPa). Higher magnifications of the medial CC (D_iii_) and the extreme lateral part of the CC (D_iv_) are illustrated in (D_ii_). Callosal axons from the medial cortical area grow in the dorsal path of the CC, whereas axons from the lateral cortex grow in the ventral path. An asterisk (*) indicates a cluster of cells in the extreme lateral part of the CC. (E–H) Immunohistochemical staining for L1 (E), Npn-1 (F), and binding site staining for ephrinA5 (G) in coronal CC slices of E18.5 mouse. (H) Double immunohistochemical labeling for DCC and GFAP on coronal CC section of P0 mice. GFAP-positive glial cells are present in the CC at the midline, the indusium griseum (IG), and the glial wedge (GW), surrounding callosal axons. Note that callosal axons originating from medial or lateral cortex express different sets of guidance molecule receptors (Npn-1 and DCC, or ephrin-A5 binding sites) and segregate in the dorsal and ventral portion of the CC, respectively. All slices were counterstained for nuclei with Hoechst. Bar indicates 1,735 µm in (D_i_), 435 µm in (A_i_ and D_ii_), 220 µm in (A_iii_, B_i_, C_i_, D_iii_, D_iv_, E, F, G, and H), and 70 µm in (A_ii_, A_iv_, B_ii_, and C_ii_). LV, lateral ventricle.
(9.83 MB TIF)

Figure S3 **GAD67-GFP-positive GABAergic interneurons and CR-positive glutamatergic neurons of the CC disappear at early postnatal ages.** (A–D) Immunohistochemical staining for CR (red) in coronal telencephalon sections from transgenic mice expressing GAD67-GFP (green) at P1 (A_i_–A_ii_), P7 (B_i_–B_ii_), P14 (C_i_–C_ii_), and P21 (D_i_–D_ii_). (A_ii_ and B_ii_) illustrate the lateral extensions of the CC seen in (Ai and Bi), respectively. (C_ii_ and D_ii_) illustrate the extreme lateral extensions of the CC seen in (C_i_ and D_i_), respectively. (A_i_–A_ii_) At P1, CR^+^ glutamatergic neuron number is already drastically reduced compared to embryonic ages, whereas GAD67-GFP^+^ neurons are still present. (B_i_–B_ii_ and C_i_–C_ii_) From P7 to P14, CR^+^ glutamatergic neurons have completely disappeared from the CC. Similarly, although a compact cluster of GAD67-GFP^+^ GABAergic interneurons remains in the extreme lateral part of the CC (*), only a few isolated GAD67-GFP^+^ neurons are detected in the medial CC. It is interesting to notice that some of the GAD67-GFP^+^ neurons present in the CC start only now to express the CR marker. (D_i_–D_ii_) At P21, GAD67-GFP^+^ GABAergic interneurons neurons have also completely disappeared in the CC. (E) Double immunohistochemical staining for CR and cleaved caspase-3 (Casp3) in coronal CC sections from P0 mice. (E_ii_) is a high-power view of the lateral CC seen in (E_i_). (F) Immunohistochemistry for cleaved caspase-3 in CC sections from GAD67-GFP transgenic mice at P7. (F_ii_) is a high-power view of GAD67-GFP/Casp3-positive neurons seen in (F_i_). (F_i_ and F_ii_) illustrate the GAD67-GFP^+^ GABAergic interneurons forming the cluster of cells in the extreme lateral part of the CC (*). Both neuronal populations of the CC appear to undergo cell death at postnatal ages since they express cleaved caspase-3. (G–H) Pre-embedding immunostaining for CR at P3 (G) or GAD67-derived GFP at P14 (H) indicate that both neuronal populations die at early postnatal ages. (G) CR-positive glutamatergic neurons in an advanced state of type II degeneration with several autophagic vacuoles observable within the cytoplasm (arrows). (H) GABAergic interneurons in the middle phase of type IIIB degeneration. The endoplasmic reticulum, Golgi apparatus, and nuclear envelope are extremely dilated (arrows). Bar indicates 220 µm in (E_i_), 110 µm in (A_i_, A_ii_, B_i_, B_ii_, C_i_, C_ii_, D_i_, and D_ii_), 50 µm in (F_i_ and E_ii_), 20 µm in (F_ii_), and 0.9 µm in (G and H). H, hippocampus; HIC, hippocampal commissure; N, nucleus.
(9.44 MB TIF)

Figure S4 **CC neurons form neuronal rows channelling callosal axons.** Semithin coronal (A) and horizontal (C) sections of E18.5 mouse CC counterstained with toluidine blue. Note the presence of multiple rows of dense neuronal clusters forming parallel trails in the CC. (B_i_–B_ii_ and D_i_–D_ii_) Electron micrographs of the ultrathin coronal CC section adjacent to the semithin section seen in (A). Callosal axons (B_ii_ and D_ii_) (arrowheads) fill in the spaces between the neurons forming a network in the CC. The walls of this neuronal network are constituted by the cell bodies and the neurites of the neurons (stars). Depending on their orientation, the neuronal barriers appear as multiple alveoli (B_i_–B_ii_) or as rows (D_i_–D_ii_) surrounding axons following parallel trajectories. (E) Illustration of the isosurfaces (E_ii_) obtained from the Mash1-GFP, CR, and Hoechst staining in (E_i_). Note the perfect match between the isosurface representation and the stainings. (E_iii_ to E_vii_) The animation mode of IMARIS 4.3 software on the isosurface files reveals the 3-D organization of the cell-free spaces within the thickness of the CC. The 3-D reconstruction is reoriented (see *X* and *Y* symbols) according to the direction of axonal growth within the CC. One of the putative entrances for callosal axons is indicated by an arrowhead in (E_iii_ and E_iv_). (E_v_–E_vii_) CR and GAD67/Mash1-GFP-positive cells are seen to form transversely oriented cell-free spaces in which callosal axons are hypothesized to grow preferentially. Bar indicates 180 µm in (A and C), 70 µm in (E_i _and E_ii_), 31 µm in (B_i_), 6.5 µm in (D_i_), and 4 µm in (B_ii_ and D_ii_). LV, lateral ventricle.
(9.76 MB TIF)

Figure S5 **Abnormal callosal axon pathfinding in** ***Mash1^−/−^*** **mice.** (A–D) Single immunohistochemistry for Satb2 (A_i_–A_ii_ and B_i_–B_ii_) and for GABA (C_i_–C_ii_ and D_i_–D_ii_) in coronal sections from E18.5 WT (A_i_–A_ii_ and C_i_–C_ii_) and *Mash1^−/−^* (B_i_–B_ii_ and D_i_–D_ii_) mice. *Mash1^−/−^* embryos exhibit a drastic reduction of GABAergic interneurons through the CC and the IG compare to WT embryos. By contrast, Satb2-positive callosal pyramidal neurons in cortical layers V–VI are not affected in the *Mash1^−/−^*. (E and F) Double immunohistochemistry for CR and Nestin in coronal CC sections from E16.5 WT (E_i_–E_ii_) and *Mash1^−/−^* (F_i_–F_ii_) mice. (E_ii_ and F_ii_) Higher power views of the Nestin staining in the glial wedge (GW) of (E_i_ and F_i_). The loss of Mash1-positive GABAergic interneurons of the CC does not cause any disorganization of glutamatergic CR^+^ neurons in the lateral part of the CC (CC, Lat) (arrowheads in [E_i_ and F_i_]). Glial cells of the indusium griseum (IG) and of the glial wedge (GW) are similar in control and *Mash1^−/−^* brains. (G and H) E18.5 CC coronal sections showing tracing of callosal axons by insertion of DiI and DiA crystals, respectively, in the frontal (CFr) and parietal (CPa) cortex of WT (G_i_–G_iv_) and *Mash1^−/−^* (H_i_–H_iv_) mice. Whereas callosal axons of the dorsal path (DiI-labeled) and of the ventral path (DiA-labeled) are segregated in the WT CC, they intermix and are misrouted in the mutant (arrowheads in [H_ii_–H_iv_]). Schematic drawings of callosal fiber trajectories in the WT (G_v_) and *Mash1^−/−^* (H_v_) brain, respectively. Bar indicates 435 µm in (A_i_, B_i_, G_i_, and H_i_), 160 µm in (A_ii_ and B_ii_), 110 µm in (C_i_, C_ii_, D_i_, D_ii_, E_i_, F_i_, G_ii_, G_iii_, G_iv_, H_ii_, H_iii_, and H_iv_), and 70 µm in (E_ii_ and F_ii_). LV, lateral ventricle; SVZ, subventricular zone.
(9.31 MB TIF)

Figure S6 **Expression of guidance factors and receptors in WT and** ***Mash1^−/−^*** **mice.** In situ hybridization for *EphA4* (A_i_–A_ii_ and B_i_–B_ii_), for *ephrinB2* (C_i_–C_ii_ and D_i_–D_ii_), for *EphB1* (E_i_–E_ii_ and F_i_–F_ii_), for *Npn1* (G_i_–G_ii_ and H_i_–H_ii_), for *Sema3C* (I_i_–I_ii_ and J_i_–J_ii_), and for *Slit2* (K_i_–K_ii_ and L_i_–L_ii_) mRNAs on coronal sections from E18.5 WT (A_i_–A_ii_, C_i_–C_ii_, E_i_–E_ii_, G_i_–G_ii_, I_i_–I_ii_, and K_i_–K_ii_) and *Mash1^−/−^* (B_i_–B_ii_, D_i_–D_ii_, F_i_–F_ii_, H_i_–H_ii_, J_i_–J_ii_, and L_i_–L_ii_) mice. Ephrins, Sema3C, and its receptor Npn1, as well as Slit2, all known to play a role in guidance of callosal axons are normally expressed in the *Mash1^−/−^* embryos. Bar indicates 600 µm in (A_ii_, B_ii_, C_i_, C_ii_, D_i_, D_ii_, E_i_, E_ii_, F_i_, F_ii_, G_ii_, H_ii_, I_ii_, J_ii_, K_i_, K_ii_, L_i_, and L_ii_) and 300 µm in (A_i_, B_i_, G_i_, H_i_, I_i_, and J_i_). CFr, frontal cortex; LV, lateral ventricle.
(9.83 MB TIF)

Figure S7 GABAergic interneurons of the CC are required for correct callosal axon navigation. (Ai) Experimental paradigm used to confirm the growth of E16.5 GFP+ WT callosal axons in slices from WT mice. To this end, GFP+ WT frontal cortex (CFr) is transplanted in a WT slice. (Aii–Aiii) GFP immunocytochemistry showing that WT GFP+ callosal axons grow normally and cross the midline when they are exposed to a WT environment. (Bi) Experimental paradigm used to test whether the presence of cortical GABAergic interneurons is necessary to direct the growth of callosal axons. To this end, GFP+ *Mash1^−/−^* CFr is transplanted in a WT slice. (Bii-Biii) GFP immunocytochemistry showing that *Mash1^−/−^* GFP+ callosal axons grow normally and cross the midline when they are exposed to a wild-type environment. (Ci) Experimental paradigm used to test whether the CC GABAergic interneurons are required for normal growth of callosal axons. To this end, GFP+ WT cortex is transplanted in a *Mash1^−/−^* slice missing GABAergic interneurons. (Cii–Ciii) GFP immunocytochemistry showing that GFP+ callosal axons of WT cortical explants do not cross the CC midline, but rather form Probst bundles (PB, open arrowhead). Bar indicates 435 µm in (Aii, Bii, and Cii) and 220 µm in (Aiii, Biii, and Ciii).
(8.57 MB TIF)

Figure S8 **Localization of Sema3C in CC development and commissure defects in** ***Sema3C^−/−^*** **mice.** (A_i_–A_ii_) In situ hybridization for *Sema3C* mRNAs on coronal CC slices of E17.5 GAD67-GFP transgenic mice. (B_i_–B_ii_) In situ hybridization for *Sema3C* mRNAs combined with immunohistochemical staining for GFAP in coronal telencephalon sections of E17.5 wild type mice. (A_ii_ and B_ii_) are higher power views of the CC seen in (A_i_ and B_i_), respectively. Within the CC, Sema3C mRNAs are never detected in GAD67-GFP^+^ GABAergic interneurons nor in GFAP^+^ astroglial cells. (C and D) Double immunohistochemistry for CR and Npn-1 (Ci and Di) and for GFAP and L1 (C_ii_ and D_ii_) in coronal CC sections from E17.5 WT (C_i_–C_ii_) and *Sema3C^−/−^* (D_i_–D_ii_) mice. (C_i_–C_ii_) At E17.5, the hemispheres of the WT brain have fused, allowing both callosal and hippocampal commissure (HIC) fibers labeled with Npn-1 and L1 to cross the midline and project into the contralateral cortex. (D_i_–D_ii_) By contrast, in *Sema3C^−/−^*, the hemispheres do not fuse properly, and the callosal fibers do not cross the midline, but instead form ectopic bundles of axons on either side of it in the IG, reminiscent of Probst bundles (PB; arrowheads in [D_i_ and D_ii_]). (C_i_ and D_i_) CR-positive neurons distribute normally in the *Sema3C^−/−^* CC. (C_ii_ and D_ii_) In control and *Sema3C^−/−^* brains, glial cells distribute similarly in the IG and GW, and extend radial processes from the lateral ventricles towards the midline. Bar indicates 220 µm in (B_i_, C_i_, C_ii_, D_i_, and D_ii_), 110 µm in (A_i_ and B_ii_), and 70 µm in (A_ii_). LV, lateral ventricle; SVZ, subventricular zone; VZ, ventricular zone.
(8.63 MB TIF)

Video S1 **The neurons of the CC cooperate to form complex neuronal network.** Video S1 was coded using DivX codec. The DivX coded for Windows can be downloaded free on the following site: http://www.divx.com/divx/windows/. Downloading this allows the viewing of video S1. Downloading video S1 can take around 30 s. The geometry of the cell-free spaces and the cellular environment of the isosurface for the three stains (CR, Mash1-GFP, and Hoechst) are explored with the navigator function of IMARIS 4.3 software. The 3-D reconstruction is reoriented according to the direction of axonal growth within the CC. Exploring the thickness of the 3-D stack imaging of the CC reveals that the CR-positive neurons (red) and the GFP-positive GABAergic interneurons (green) form adjacent rows of neurons regularly distributed within the entire CC. Rows of neurons are separated by cell-free domains of small or large diameter, which likely segregate the callosal axons into bundles. This network of cell-free domain could define preferred pathways for the navigation of callosal axons. This analysis shows that the two types of neurons that transiently populate the CC form a complex neuronal network and are well positioned to interact with ingrowing callosal axons.
(10.00 MB AVI)

## Abbreviations

AgCC: agenesis of the corpus callosum
CC: corpus callosum
CCi: cingulate cortex
E: embryonic day
GW: glial wedge
IG: indusium griseum
IZ: intermediate zone
LGE: lateral ganglionic eminence
MGE: medial ganglionic eminence
P: postnatal day
SEP: septum
siRNA: small interfering RNA
WT: wild-type
