# Supplementary figures and images for: Transient Neuronal Populations Are Required to Guide Callosal Axons: A Role for Semaphorin 3C
Source: PLoS Biol. 2009 Oct 27;7(10):e1000230. doi: 10.1371/journal.pbio.1000230 (PMC2762166; doi:10.1371/journal.pbio.1000230)

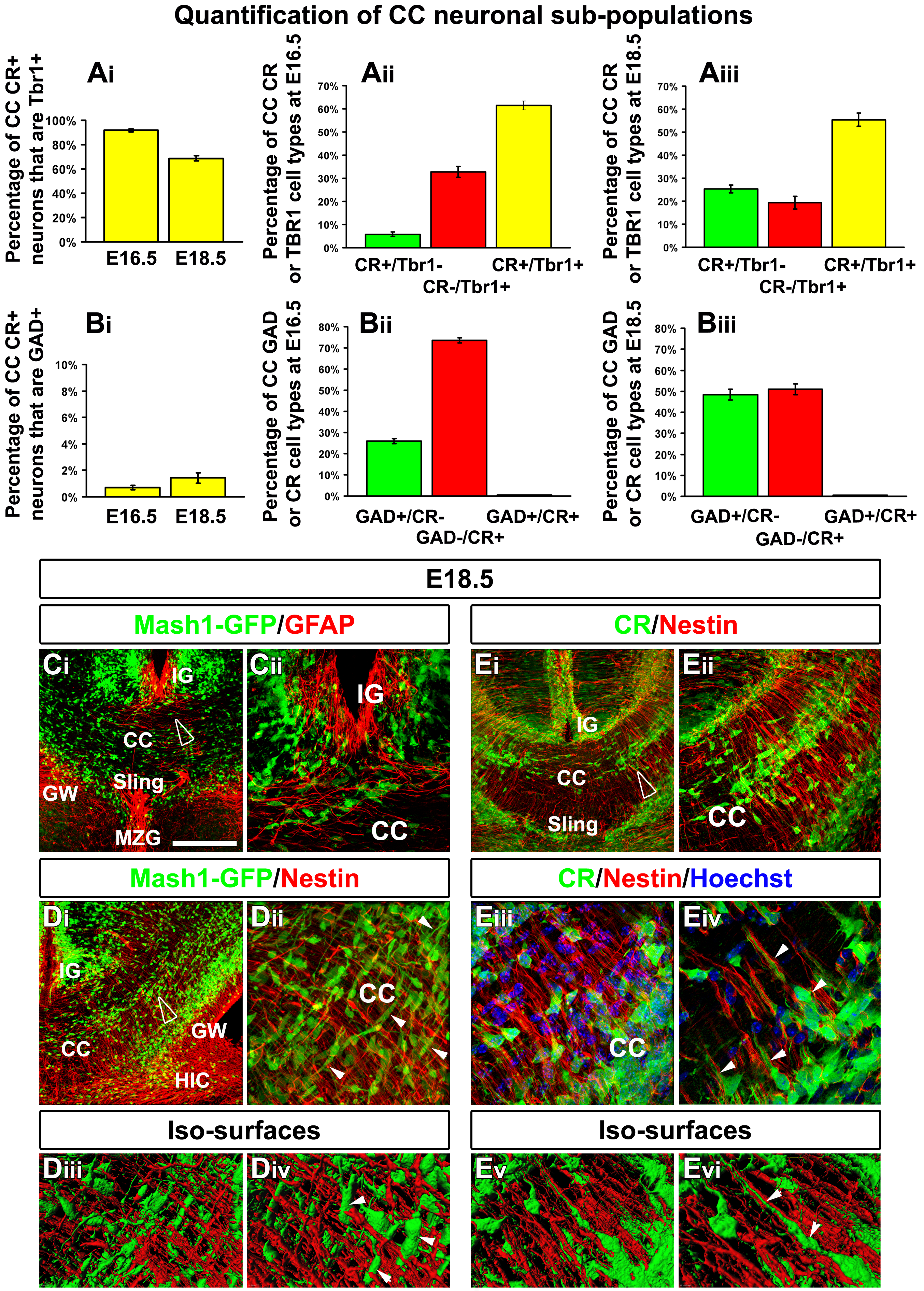

Supplement: Figure S1 — CC neuronal subpopulations and complementary organization of CC neurons and astroglial cells during embryonic development. (Ai) Bars (means±standard error of the mean [SEM] from a sample of 1,415 CR+ neurons at E16.5 and 252 CR+ neurons at E18.5) represent the percentage of CC CR+ neurons expressing Tbr1. (Aii and Aiii) Repartition of Tbr1+ and CR+ neuronal populations of the CC IZ at E16.5 (Aii) and E18.5 (Aiii). Bars (means±SEM from a sample of 2,108 neurons in [Aii] and 312 in [Aiii]) represent the percentage of neurons expressing or not Tbr1 and CR compared to the total number of neurons of the CC labeled with Tbr1 and/or CR. (Bi) Bars (means±SEM from a sample of 1,907 CR+ neurons at E16.5 and 1,898 CR+ neurons at E18.5) represent the percentage of CC CR+ neurons expressing the GAD67-GFP. (Bii and Biii) Repartition of GAD67-GFP+ and CR+ neuronal populations of the CC IZ at E16.5 (Bii) and E18.5 (Biii). Bars (means±SEM from a sample of 2,580 neurons in [Bii] and 3,442 in [Biii]) represent the percentage of neurons expressing or not the GAD67-GFP and the CR compared to the total number of neurons of the CC labeled with GAD67-GFP and/or CR. (C and D) Immunohistochemical staining for GFAP (Ci–Cii) and nestin (Di–Dii) in coronal telencephalon sections from mice expressing Mash1-GFP at E18.5. (Cii and Dii) are higher power views of the portion of the CC seen in (Ci and Di) (open arrowheads), respectively. (Diii–Div) Illustration of the isosurfaces obtained from the Mash1-GFP and nestin staining in (Dii). Mash1-GFP GABAergic interneurons (arrowheads) migrate tangentially within the CC through radial glial processes that extend from the ventricular zone to the cortical marginal zone. (E) Double immunohistochemistry for CR and nestin (Ei–Eiv) in coronal sections from E18.5 mice. (Eii–Eiv) are higher power views of the CC seen in (Ei) (open arrowhead) and (Ev–Evi) are isosurface illustrations obtained from the CR and nestin staining in Eiii. CR-positive neurons (arrowhead [file pbio.1000230.s001.tif]

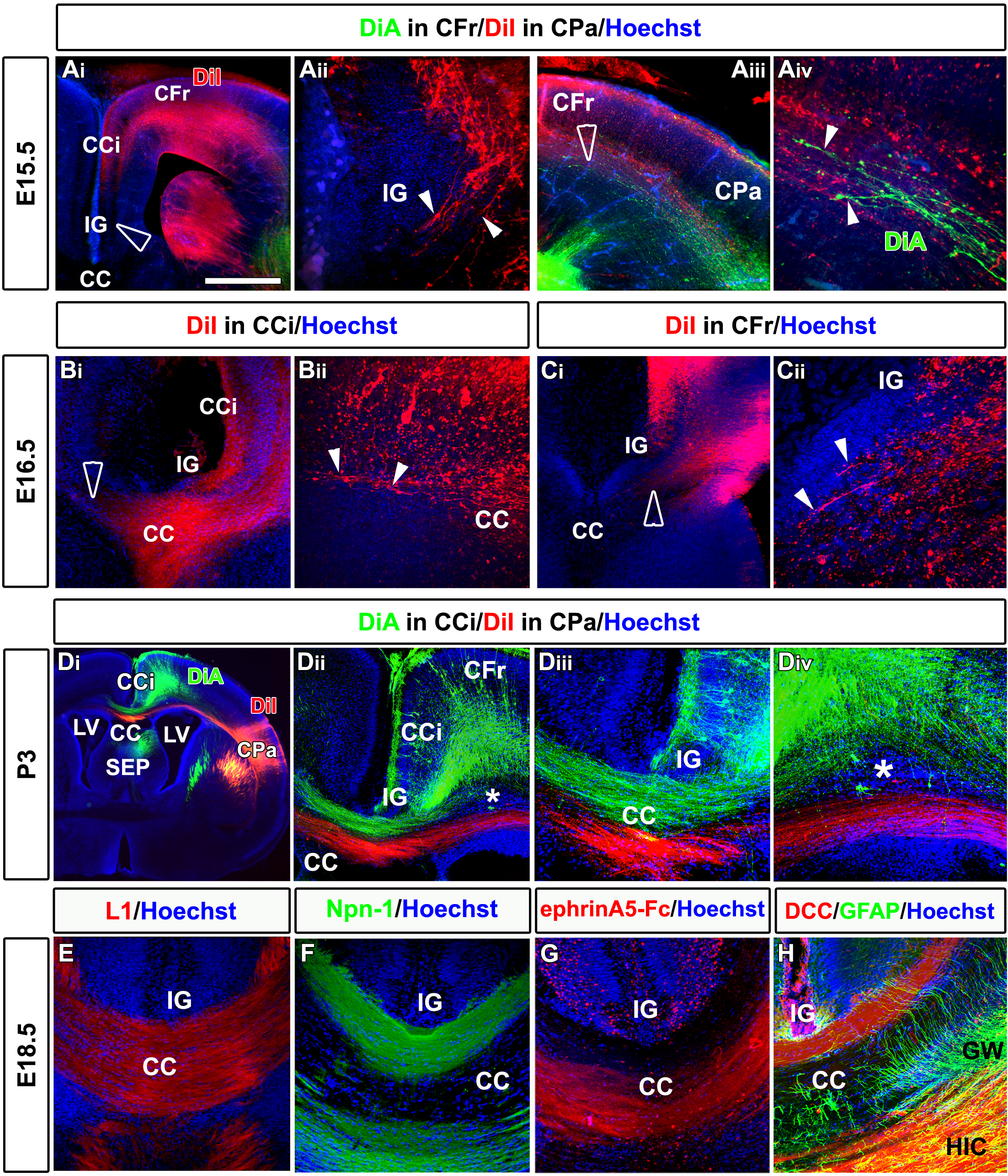

Supplement: Figure S2 — Callosal axons exhibit a dorsoventral topographic organization within the CC. (A) Coronal sections from E15.5 mice showing callosal axons labeled by insertion of DiI and DiA crystals, respectively, in the frontal (CFr) and parietal (CPa) cortices. Higher magnifications of the CC (Aii) and of the lateral white matter (Aiv) are illustrated, respectively, in (Ai and Aiii) (open arrowheads). Arrowheads in (Aii and Aiv) point to callosal axon endings. Callosal axons from the frontal cortex have not yet reached the future CC region at E15.5, whereas the callosal axons from the parietal cortex are growing through the white matter bellow the frontal cortex. (B and C) Coronal sections from E16.5 mice showing callosal axons labeled by insertion of DiI in the cingulate (CCi) (Bi–Bii) and frontal (Ci–Cii) cortices. Higher magnifications of the CC (Bii and Cii) are illustrated, respectively, in (Bi and Ci) (open arrowheads). Arrowheads in (Bii and Cii) point to callosal axon endings. At E16.5, although pioneer callosal axons from the CCi have already crossed the CC midline, the callosal axons from the frontal cortex have not yet reached the CC midline. (Di–Div) Coronal sections from P3 mice showing callosal axons labeled by insertion of DiA and DiI crystals, respectively, in the frontal cortex (CFr) and the parietal cortex (CPa). Higher magnifications of the medial CC (Diii) and the extreme lateral part of the CC (Div) are illustrated in (Dii). Callosal axons from the medial cortical area grow in the dorsal path of the CC, whereas axons from the lateral cortex grow in the ventral path. An asterisk (*) indicates a cluster of cells in the extreme lateral part of the CC. (E–H) Immunohistochemical staining for L1 (E), Npn-1 (F), and binding site staining for ephrinA5 (G) in coronal CC slices of E18.5 mouse. (H) Double immunohistochemical labeling for DCC and GFAP on coronal CC section of P0 mice. GFAP-positive glial cells are present in the CC at the midline, the indusium griseum (I [file pbio.1000230.s002.tif]

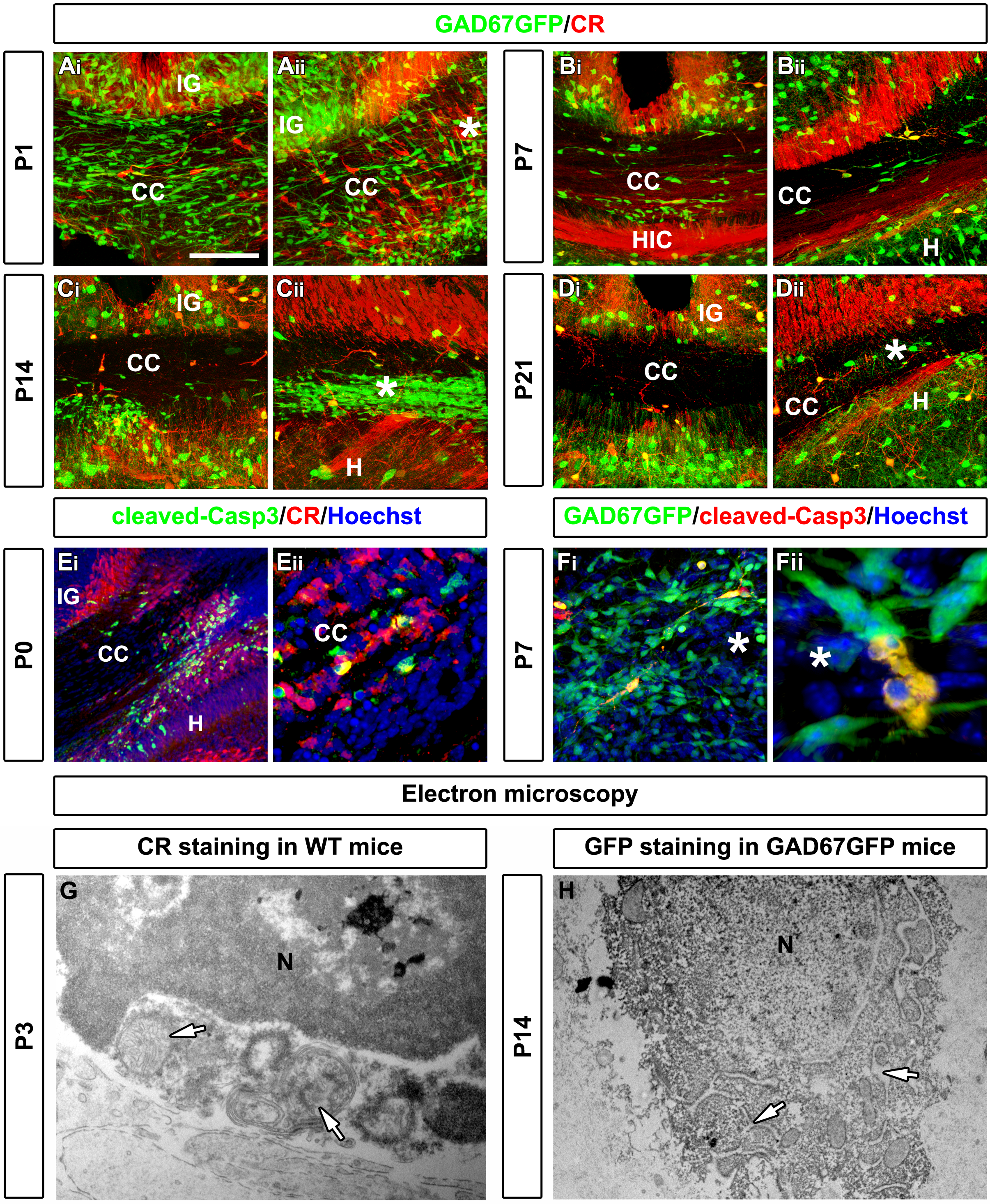

Supplement: Figure S3 — GAD67-GFP-positive GABAergic interneurons and CR-positive glutamatergic neurons of the CC disappear at early postnatal ages. (A–D) Immunohistochemical staining for CR (red) in coronal telencephalon sections from transgenic mice expressing GAD67-GFP (green) at P1 (Ai–Aii), P7 (Bi–Bii), P14 (Ci–Cii), and P21 (Di–Dii). (Aii and Bii) illustrate the lateral extensions of the CC seen in (Ai and Bi), respectively. (Cii and Dii) illustrate the extreme lateral extensions of the CC seen in (Ci and Di), respectively. (Ai–Aii) At P1, CR+ glutamatergic neuron number is already drastically reduced compared to embryonic ages, whereas GAD67-GFP+ neurons are still present. (Bi–Bii and Ci–Cii) From P7 to P14, CR+ glutamatergic neurons have completely disappeared from the CC. Similarly, although a compact cluster of GAD67-GFP+ GABAergic interneurons remains in the extreme lateral part of the CC (*), only a few isolated GAD67-GFP+ neurons are detected in the medial CC. It is interesting to notice that some of the GAD67-GFP+ neurons present in the CC start only now to express the CR marker. (Di–Dii) At P21, GAD67-GFP+ GABAergic interneurons neurons have also completely disappeared in the CC. (E) Double immunohistochemical staining for CR and cleaved caspase-3 (Casp3) in coronal CC sections from P0 mice. (Eii) is a high-power view of the lateral CC seen in (Ei). (F) Immunohistochemistry for cleaved caspase-3 in CC sections from GAD67-GFP transgenic mice at P7. (Fii) is a high-power view of GAD67-GFP/Casp3-positive neurons seen in (Fi). (Fi and Fii) illustrate the GAD67-GFP+ GABAergic interneurons forming the cluster of cells in the extreme lateral part of the CC (*). Both neuronal populations of the CC appear to undergo cell death at postnatal ages since they express cleaved caspase-3. (G–H) Pre-embedding immunostaining for CR at P3 (G) or GAD67-derived GFP at P14 (H) indicate that both neuronal populations die at early postnatal ages. (G) CR-positive glutamatergic neurons in an advanced [file pbio.1000230.s003.tif]

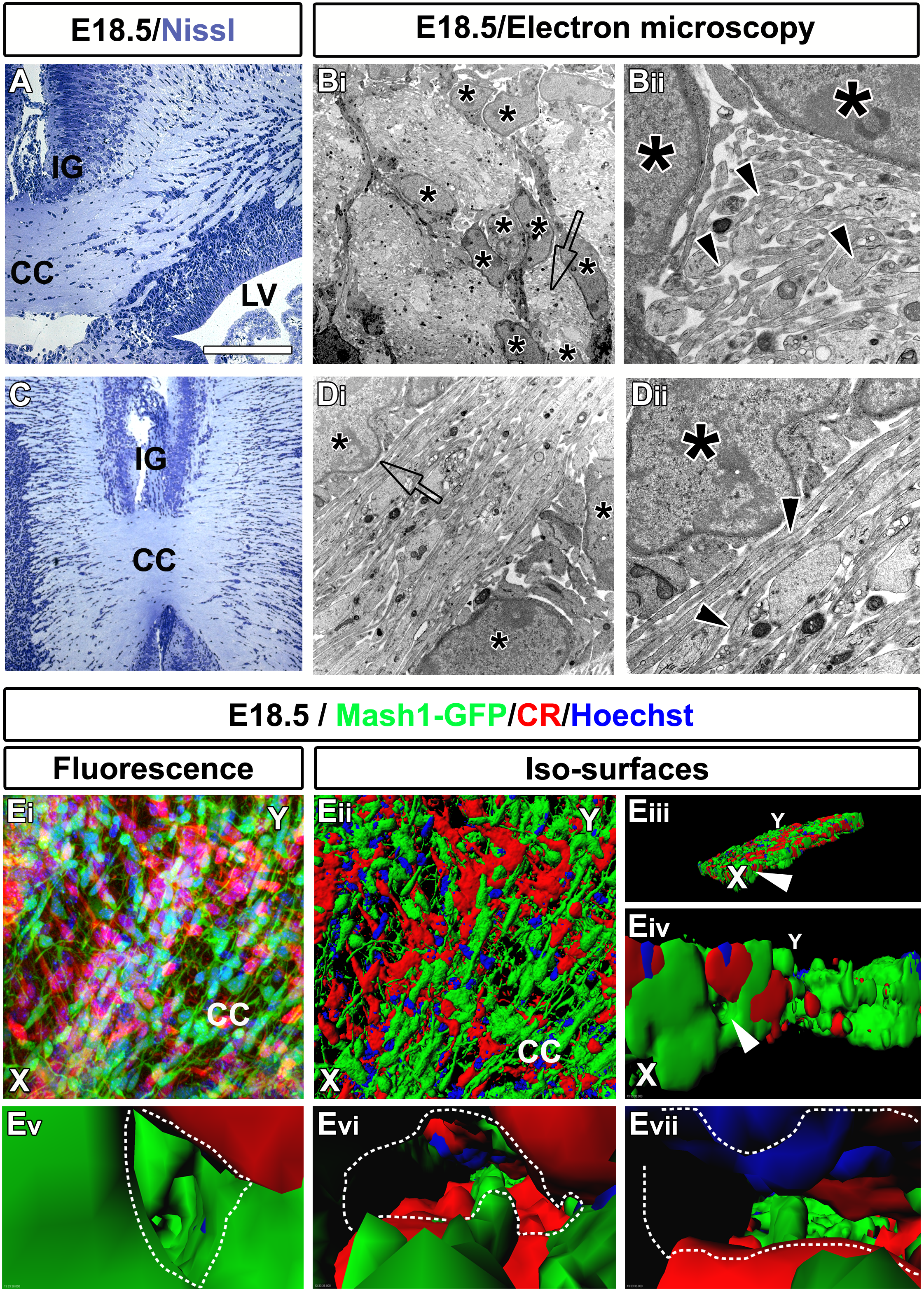

Supplement: Figure S4 — CC neurons form neuronal rows channelling callosal axons. Semithin coronal (A) and horizontal (C) sections of E18.5 mouse CC counterstained with toluidine blue. Note the presence of multiple rows of dense neuronal clusters forming parallel trails in the CC. (Bi–Bii and Di–Dii) Electron micrographs of the ultrathin coronal CC section adjacent to the semithin section seen in (A). Callosal axons (Bii and Dii) (arrowheads) fill in the spaces between the neurons forming a network in the CC. The walls of this neuronal network are constituted by the cell bodies and the neurites of the neurons (stars). Depending on their orientation, the neuronal barriers appear as multiple alveoli (Bi–Bii) or as rows (Di–Dii) surrounding axons following parallel trajectories. (E) Illustration of the isosurfaces (Eii) obtained from the Mash1-GFP, CR, and Hoechst staining in (Ei). Note the perfect match between the isosurface representation and the stainings. (Eiii to Evii) The animation mode of IMARIS 4.3 software on the isosurface files reveals the 3-D organization of the cell-free spaces within the thickness of the CC. The 3-D reconstruction is reoriented (see X and Y symbols) according to the direction of axonal growth within the CC. One of the putative entrances for callosal axons is indicated by an arrowhead in (Eiii and Eiv). (Ev–Evii) CR and GAD67/Mash1-GFP-positive cells are seen to form transversely oriented cell-free spaces in which callosal axons are hypothesized to grow preferentially. Bar indicates 180 µm in (A and C), 70 µm in (Ei and Eii), 31 µm in (Bi), 6.5 µm in (Di), and 4 µm in (Bii and Dii). LV, lateral ventricle. (9.76 MB TIF) [file pbio.1000230.s004.tif]

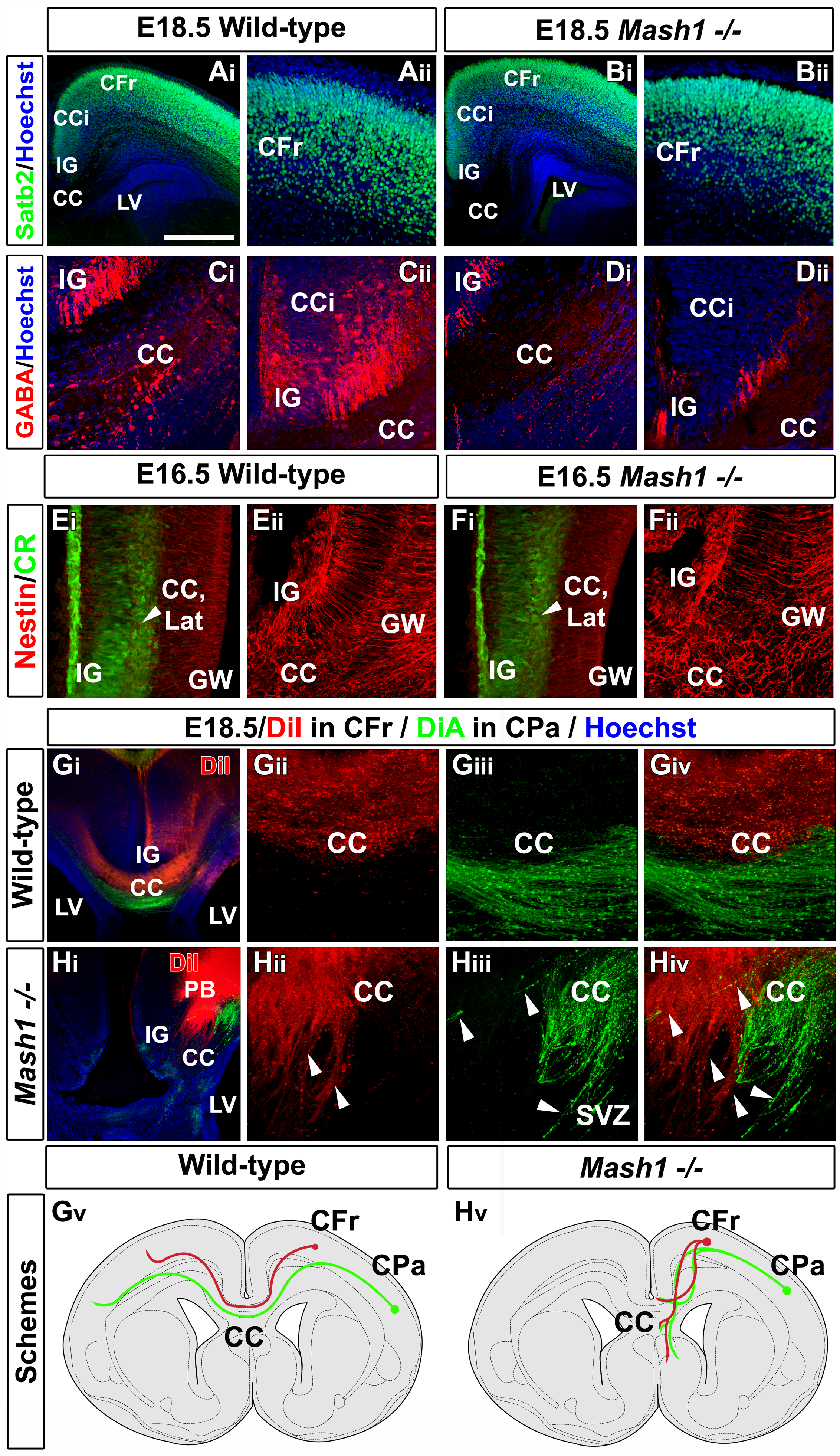

Supplement: Figure S5 — Abnormal callosal axon pathfinding in Mash1−/− mice. (A–D) Single immunohistochemistry for Satb2 (Ai–Aii and Bi–Bii) and for GABA (Ci–Cii and Di–Dii) in coronal sections from E18.5 WT (Ai–Aii and Ci–Cii) and Mash1−/− (Bi–Bii and Di–Dii) mice. Mash1−/− embryos exhibit a drastic reduction of GABAergic interneurons through the CC and the IG compare to WT embryos. By contrast, Satb2-positive callosal pyramidal neurons in cortical layers V–VI are not affected in the Mash1−/−. (E and F) Double immunohistochemistry for CR and Nestin in coronal CC sections from E16.5 WT (Ei–Eii) and Mash1−/− (Fi–Fii) mice. (Eii and Fii) Higher power views of the Nestin staining in the glial wedge (GW) of (Ei and Fi). The loss of Mash1-positive GABAergic interneurons of the CC does not cause any disorganization of glutamatergic CR+ neurons in the lateral part of the CC (CC, Lat) (arrowheads in [Ei and Fi]). Glial cells of the indusium griseum (IG) and of the glial wedge (GW) are similar in control and Mash1−/− brains. (G and H) E18.5 CC coronal sections showing tracing of callosal axons by insertion of DiI and DiA crystals, respectively, in the frontal (CFr) and parietal (CPa) cortex of WT (Gi–Giv) and Mash1−/− (Hi–Hiv) mice. Whereas callosal axons of the dorsal path (DiI-labeled) and of the ventral path (DiA-labeled) are segregated in the WT CC, they intermix and are misrouted in the mutant (arrowheads in [Hii–Hiv]). Schematic drawings of callosal fiber trajectories in the WT (Gv) and Mash1−/− (Hv) brain, respectively. Bar indicates 435 µm in (Ai, Bi, Gi, and Hi), 160 µm in (Aii and Bii), 110 µm in (Ci, Cii, Di, Dii, Ei, Fi, Gii, Giii, Giv, Hii, Hiii, and Hiv), and 70 µm in (Eii and Fii). LV, lateral ventricle; SVZ, subventricular zone. (9.31 MB TIF) [file pbio.1000230.s005.tif]

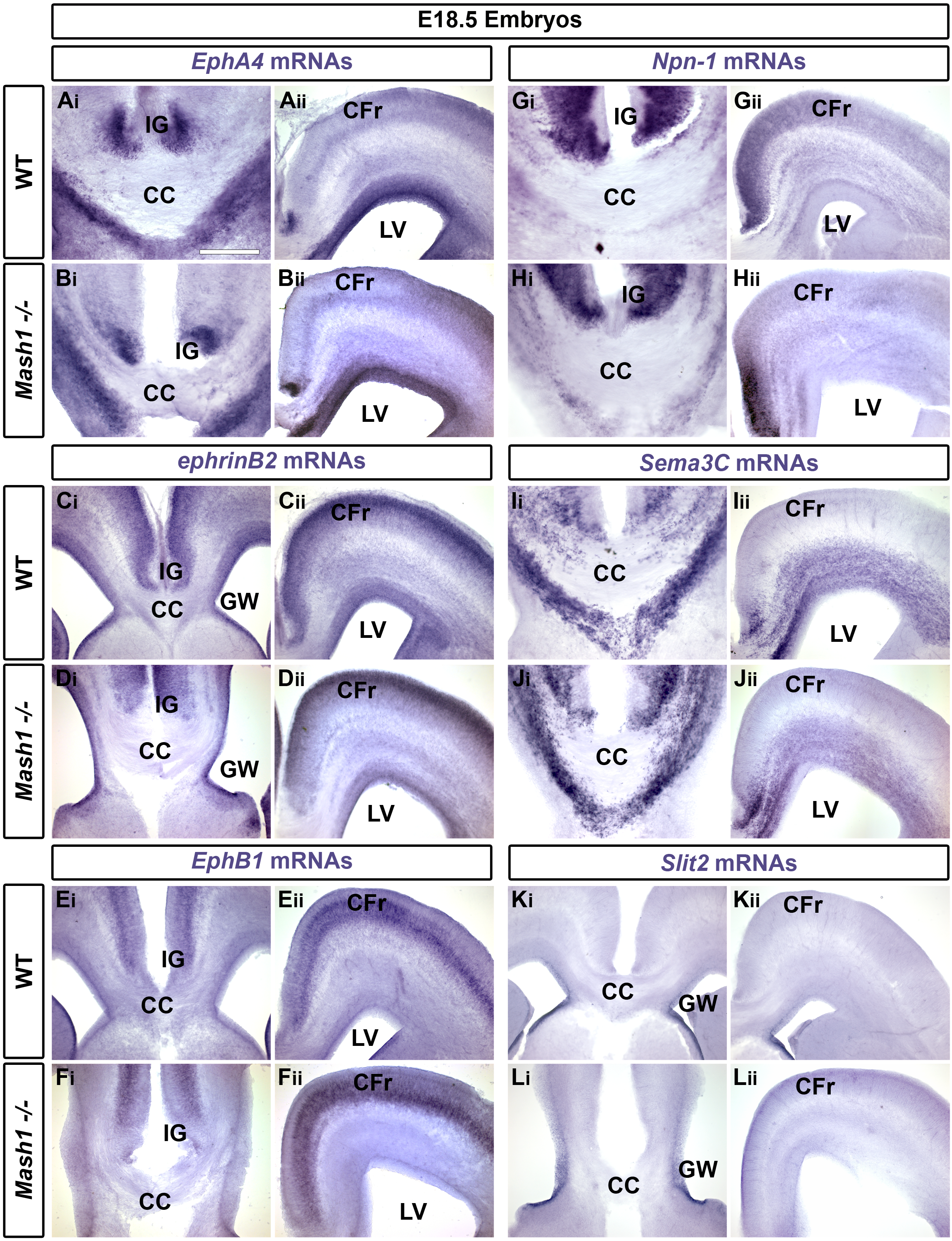

Supplement: Figure S6 — Expression of guidance factors and receptors in WT and Mash1−/− mice. In situ hybridization for EphA4 (Ai–Aii and Bi–Bii), for ephrinB2 (Ci–Cii and Di–Dii), for EphB1 (Ei–Eii and Fi–Fii), for Npn1 (Gi–Gii and Hi–Hii), for Sema3C (Ii–Iii and Ji–Jii), and for Slit2 (Ki–Kii and Li–Lii) mRNAs on coronal sections from E18.5 WT (Ai–Aii, Ci–Cii, Ei–Eii, Gi–Gii, Ii–Iii, and Ki–Kii) and Mash1−/− (Bi–Bii, Di–Dii, Fi–Fii, Hi–Hii, Ji–Jii, and Li–Lii) mice. Ephrins, Sema3C, and its receptor Npn1, as well as Slit2, all known to play a role in guidance of callosal axons are normally expressed in the Mash1−/− embryos. Bar indicates 600 µm in (Aii, Bii, Ci, Cii, Di, Dii, Ei, Eii, Fi, Fii, Gii, Hii, Iii, Jii, Ki, Kii, Li, and Lii) and 300 µm in (Ai, Bi, Gi, Hi, Ii, and Ji). CFr, frontal cortex; LV, lateral ventricle. (9.83 MB TIF) [file pbio.1000230.s006.tif]

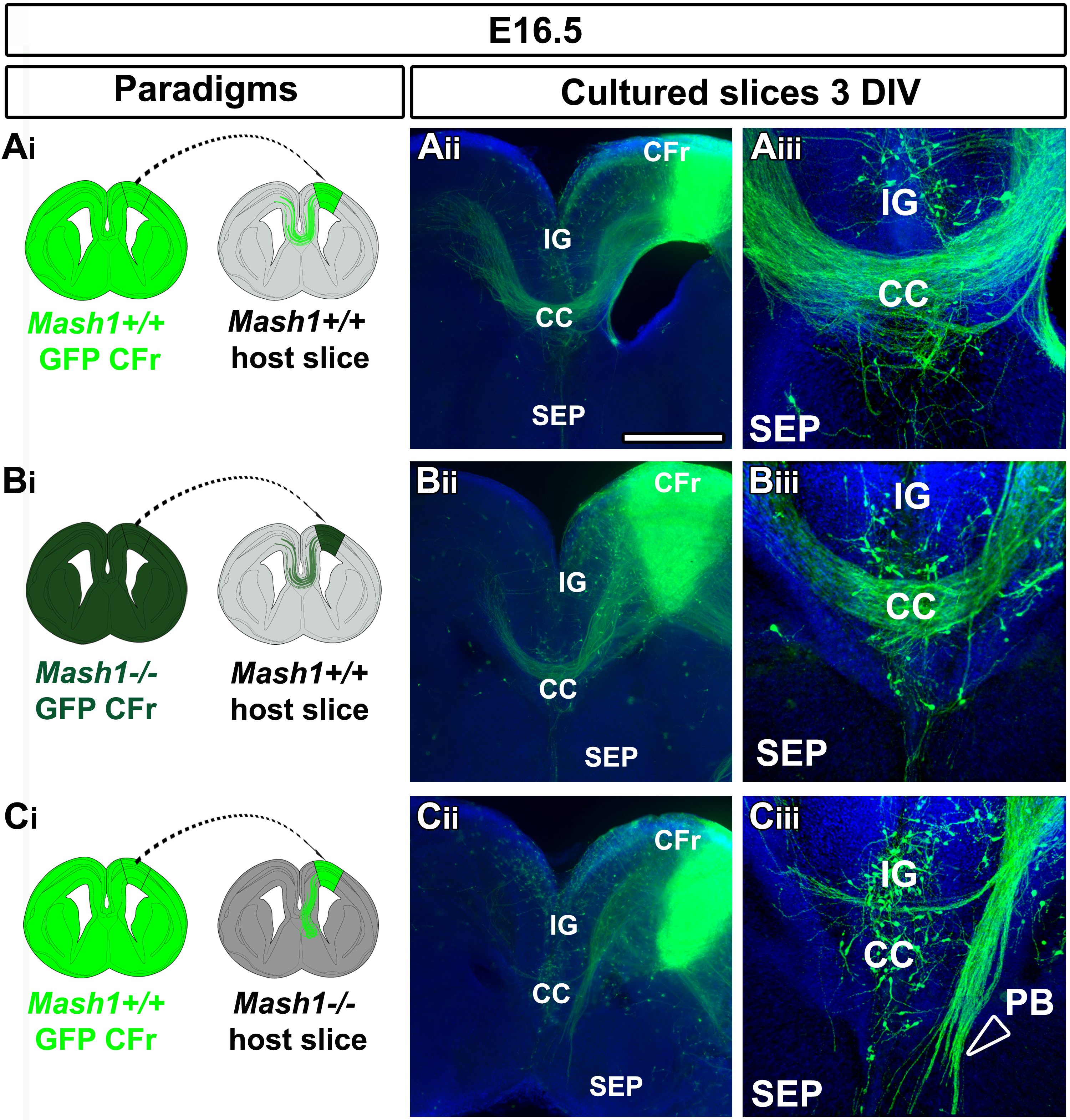

Supplement: Figure S7 — GABAergic interneurons of the CC are required for correct callosal axon navigation. (Ai) Experimental paradigm used to confirm the growth of E16.5 GFP+ WT callosal axons in slices from WT mice. To this end, GFP+ WT frontal cortex (CFr) is transplanted in a WT slice. (Aii–Aiii) GFP immunocytochemistry showing that WT GFP+ callosal axons grow normally and cross the midline when they are exposed to a WT environment. (Bi) Experimental paradigm used to test whether the presence of cortical GABAergic interneurons is necessary to direct the growth of callosal axons. To this end, GFP+ Mash1−/− CFr is transplanted in a WT slice. (Bii-Biii) GFP immunocytochemistry showing that Mash1−/− GFP+ callosal axons grow normally and cross the midline when they are exposed to a wild-type environment. (Ci) Experimental paradigm used to test whether the CC GABAergic interneurons are required for normal growth of callosal axons. To this end, GFP+ WT cortex is transplanted in a Mash1−/− slice missing GABAergic interneurons. (Cii–Ciii) GFP immunocytochemistry showing that GFP+ callosal axons of WT cortical explants do not cross the CC midline, but rather form Probst bundles (PB, open arrowhead). Bar indicates 435 µm in (Aii, Bii, and Cii) and 220 µm in (Aiii, Biii, and Ciii). (8.57 MB TIF) [file pbio.1000230.s007.tif]

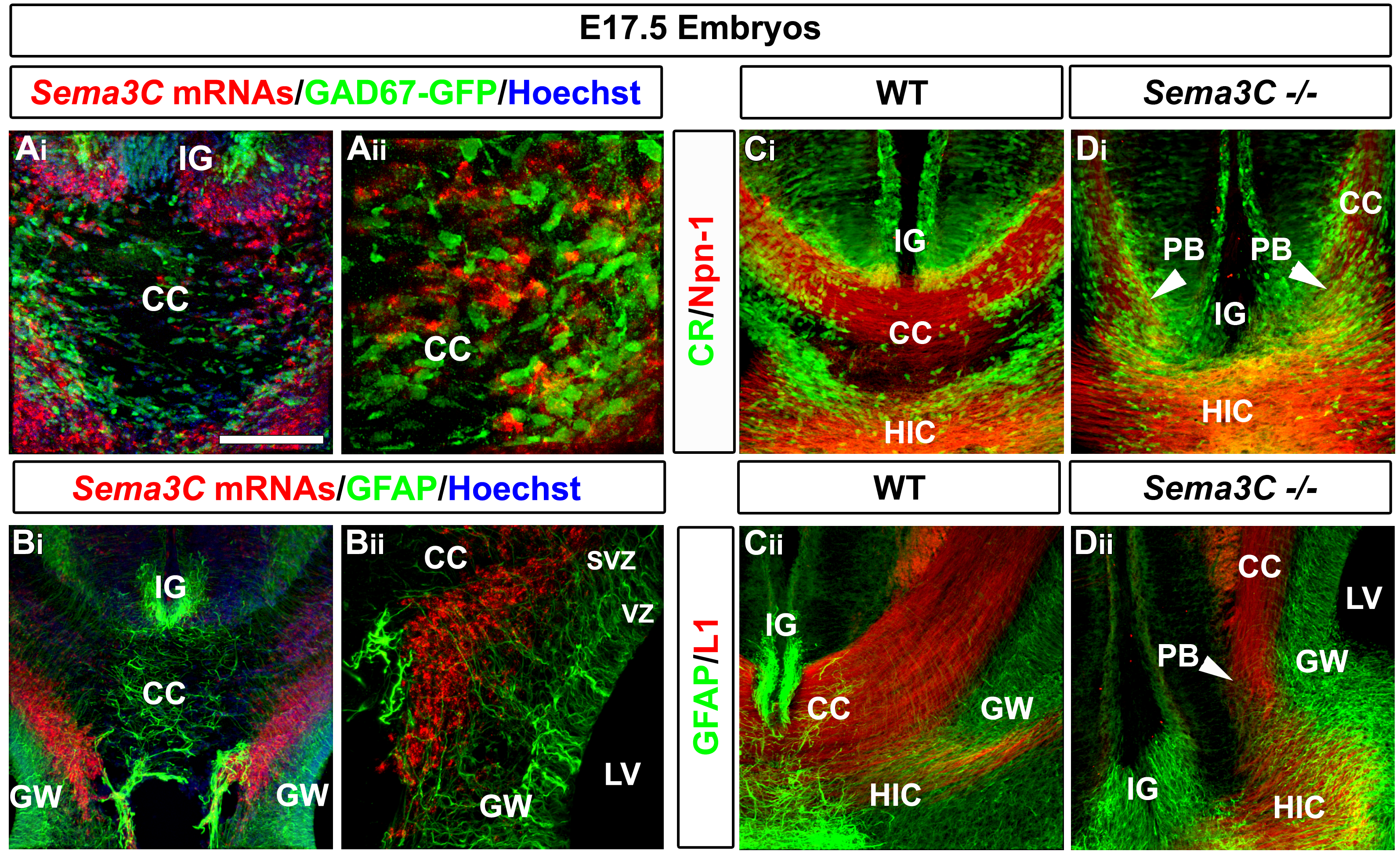

Supplement: Figure S8 — Localization of Sema3C in CC development and commissure defects in Sema3C−/− mice. (Ai–Aii) In situ hybridization for Sema3C mRNAs on coronal CC slices of E17.5 GAD67-GFP transgenic mice. (Bi–Bii) In situ hybridization for Sema3C mRNAs combined with immunohistochemical staining for GFAP in coronal telencephalon sections of E17.5 wild type mice. (Aii and Bii) are higher power views of the CC seen in (Ai and Bi), respectively. Within the CC, Sema3C mRNAs are never detected in GAD67-GFP+ GABAergic interneurons nor in GFAP+ astroglial cells. (C and D) Double immunohistochemistry for CR and Npn-1 (Ci and Di) and for GFAP and L1 (Cii and Dii) in coronal CC sections from E17.5 WT (Ci–Cii) and Sema3C−/− (Di–Dii) mice. (Ci–Cii) At E17.5, the hemispheres of the WT brain have fused, allowing both callosal and hippocampal commissure (HIC) fibers labeled with Npn-1 and L1 to cross the midline and project into the contralateral cortex. (Di–Dii) By contrast, in Sema3C−/−, the hemispheres do not fuse properly, and the callosal fibers do not cross the midline, but instead form ectopic bundles of axons on either side of it in the IG, reminiscent of Probst bundles (PB; arrowheads in [Di and Dii]). (Ci and Di) CR-positive neurons distribute normally in the Sema3C−/− CC. (Cii and Dii) In control and Sema3C−/− brains, glial cells distribute similarly in the IG and GW, and extend radial processes from the lateral ventricles towards the midline. Bar indicates 220 µm in (Bi, Ci, Cii, Di, and Dii), 110 µm in (Ai and Bii), and 70 µm in (Aii). LV, lateral ventricle; SVZ, subventricular zone; VZ, ventricular zone. (8.63 MB TIF) [file pbio.1000230.s008.tif]
